# Truncating variants reveal functional divergence of the SAMD9 and SAMD9L paralogues

**DOI:** 10.70962/jhi.20260095

**Published:** 2026-11-10

**Authors:** Petar Mitev, Aditi Khurana, Caroline Eriksson, Elisa Saccon, Saeed Eshtad, Sofie Vonlanthen, Bianca Tesi, Elham Dadfar, AnnaCarin Horne, Elisabeth Mellström, Martin Paucar, Mikael Sundin, Yenan T. Bryceson

**Affiliations:** 1 Department of Medicine Huddinge, Center for Hematology and Regenerative Medicine, Karolinska Institutet, Stockholm, Sweden; 2 Division of Biomolecular and Cellular Medicine, Department of Laboratory Medicine, Karolinska Institutet, Stockholm, Sweden; 3 Division of Clinical Immunology and Transfusion Medicine, Karolinska University Hospital, Stockholm, Sweden; 4 Division of Clinical Genetics and Genomics, Karolinska University Hospital, Stockholm, Sweden; 5 Department of Biomedical and Clinical Sciences, Linköping University, Linköping, Sweden; 6 Crown Princess Victoria’s Child and Youth Hospital, Linköping University Hospital, Linköping, Sweden; 7 Sweden and Division of Paediatric Rheumatology, Department of Women’s and Children’s Health, Karolinska Institute, Solna, Astrid Lindgren Children’s Hospital, Karolinska University Hospital, Stockholm, Sweden; 8 Department of Pediatrics, Sahlgrenska Center for Cancer Research, Institute of Clinical Sciences, Sahlgrenska Academy at University of Gothenburg, Gothenburg, Sweden; 9 Sweden and Childhood Cancer Centre, Queen Silvia Children’s Hospital, Sahlgrenska University Hospital, Gothenburg, Sweden; 10 Department of Clinical Neuroscience, Karolinska Institutet, Stockholm, Sweden; 11 Department of Clinical Science, Intervention and Technology, Karolinska Institutet, Stockholm, Sweden; 12 Division of Pediatrics, Section of Pediatric Hematology, Immunology and HCT, Astrid Lindgren Children’s Hospital, Karolinska University Hospital, Stockholm, Sweden; 13 Broegelmann Research Laboratory, Department of Clinical Science, University of Bergen, Bergen, Norway

## Abstract

Germline gain-of-function (GoF) variants in the paralogous sterile alpha motif domain containing 9 (*SAMD9*) and SAMD9-like (*SAMD9L*) genes cause myelodysplasia, primary immunodeficiencies, and neurological disorders. Predicting the functional consequences of individual variants, however, remains challenging, owing partly to the complexity of these large, multidomain proteins. Here, we functionally characterized several rare patient-derived *SAMD9/9L* variants, identifying multiple novel GoF and loss-of-function (LoF) variants, including *SAMD9L* p.C883X—the shortest documented *SAMD9L* GoF truncation within the NTPase domain. *SAMD9L* NTPase domain truncations are associated with severe, early-onset pathology, yet despite the high structural and sequence similarity between the two proteins, analogous truncating variants have not been reported in *SAMD9* patients. To examine whether this reflects a fundamental difference, we generated multiple homologous *SAMD9* truncations, which, unlike their *SAMD9L* counterparts, exhibited LoF rather than GoF activity. Additionally, established computational pathogenicity predictors failed to identify pathogenic variants or distinguish GoF from LoF. Collectively, these results demonstrate that functional outcomes in *SAMD9/9L* cannot be reliably inferred from sequence, structure, or computational prediction alone, underscoring the importance of empirical characterization.

## Teaser

Characterization of novel patient-derived SAMD9 and SAMD9L variants, including the SAMD9L p.C883X truncation, prompted systematic investigation of homologous SAMD9 truncations, which uncovered unexpected functional divergence between the two paralogues, underscoring the importance of empirical validation for accurate variant classification.

## Introduction

Germline variants in the paralogous sterile alpha motif (SAM) domain containing 9 (*SAMD9*) and SAMD9-like (*SAMD9L*) genes give rise to a remarkably diverse array of clinical presentations (1). In particular, heterozygous de novo gain-of-function (GoF) variants underlie early-onset *SAMD9*-associated MIRAGE syndrome (myelodysplasia, infection, restriction of growth, adrenal hypoplasia, genital phenotypes, and enteropathy) (2, 3) and *SAMD9L*-associated autoinflammatory disease (SAAD) (4, 5, 6), whereas heterozygous GoF variants in *SAMD9L* associated with ataxia-pancytopenia syndrome (ATXPC) with variable penetrance are typically inherited (7, 8). On the other end of the functional spectrum, autosomal recessive *SAMD9* loss-of-function (LoF) variants cause normophosphatemic familial tumoral calcinosis (9, 10, 11), while germline heterozygous *SAMD9* and *SAMD9L* LoF variants have also been identified in a subset of adult patients with myeloid neoplasms (12), which would be consistent with a tumor suppressor role supported by mouse models of *Samd9l* haploinsufficiency (13). Given the distinct clinical implications of GoF and LoF variants, accurate functional classification of *SAMD9* and *SAMD9L* variants therefore remains essential for both diagnosis and clinical management (1, 14, 15, 16).

*SAMD9* and *SAMD9L*, thought to have arisen from a gene duplication event (17, 18), are positioned in a head-to-tail orientation on human chromosome 7q and encode, through a single exon each, large cytoplasmic proteins of ∼1,600 amino acids (19) that share roughly 60% sequence identity (18). Their consensus domain architecture features, from the N terminus: a SAM, an Alba domain possessing endoribonucleolytic activity, a sirtuin 2 domain, a large P-loop NTPase domain, tetratricopeptide repeats, and an oligonucleotide/oligosaccharide-binding fold (17). Upon activation, the SAMD9 and SAMD9L Alba domains cleave tRNA^Phe^, blocking protein synthesis and inducing cell cycle arrest; GoF variants constitutively engage this mechanism, resulting in pathological growth suppression (20). The severity of this cellular effect is reflected in the strong selective pressure for somatic reversion, as carriers of GoF variants frequently acquire secondary LoF mutations or chromosomal alterations that remove the pathogenic allele. Such chromosomal rearrangements can result in monosomy 7, a chromosomal aberration associated with the development of myeloid malignancies with poor prognosis (16, 21, 22).

Distinguishing *SAMD9 and SAMD9L* GoF from LoF variants in the clinic remains a considerable challenge. While early disease onset has traditionally been taken as suggestive of GoF activity, this correlation is imperfect: GoF variants can manifest later in life with variable expressivity, and conversely, young patients presenting with *SAMD9/SAMD9L* syndrome-like symptoms may carry variants that are weak or lack GoF activity altogether, while LoF variants typically come to clinical attention only in adulthood and may exhibit very low penetrance (1). For truncating variants, position-dependent effects add another layer of complexity: while very early truncations predictably result in LoF, truncations within a specific region of the *SAMD9L* P-loop NTPase domain (approximately F886–Y950) yield a strong GoF phenotype, often associated with early-onset SAAD (5, 6, 23). Yet, despite the close structural and sequence similarity between these paralogues, whether analogous truncations in *SAMD9* similarly confer GoF activity has not been examined.

Here, we functionally characterized patient-derived *SAMD9* and *SAMD9L* variants using stable doxycycline-inducible cell lines and a flow cytometry–based 5-ethynyl-2′-deoxyuridine (EdU) incorporation assay with a custom computational pipeline. Building on our findings, we systematically investigated whether truncations in the *SAMD9* P-loop NTPase domain behave similarly to their pathogenic *SAMD9L* counterparts and demonstrate that they do not—revealing an unanticipated functional divergence between these structurally similar paralogues. Our results underscore the indispensability of empirical testing for accurate variant interpretation.

## Results

### Clinical presentation of patients with rare *SAMD9* and *SAMD9L* variants

**Figure S1.**
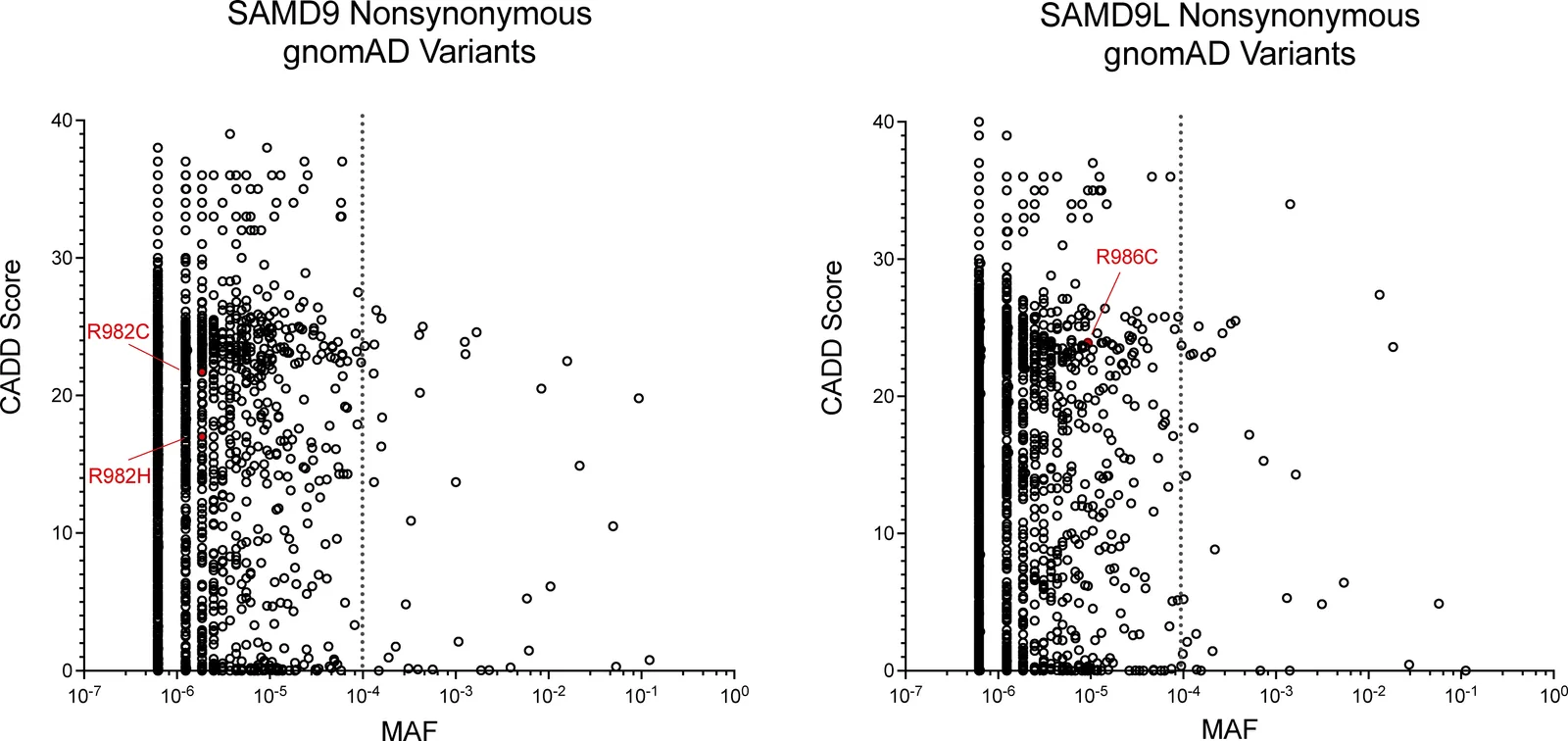
**CADD scores and population frequencies of nonsynonymous gnomAD variants in SAMD9 and SAMD9L.** CADD phred scores plotted against minor allele frequency (MAF) for all nonsynonymous SAMD9 (left) and SAMD9L (right) variants present in the gnomAD database. Established GoF variants R982C/R982H (SAMD9) and R986C (SAMD9L) are annotated. The dashed vertical lines indicate a MAF threshold of 10^−4^.

In routine clinical genome sequencing of patients with suspected autoinflammatory disease, inborn errors of immunity, bone marrow failure, or ataxia syndrome, nine rare, predicted damaging *SAMD9* and *SAMD9L* variants were identified and selected for functional characterization (Table 1 and Table S1). We used a cutoff of population minor allele frequency below 0.0001 (gnomAD v.4.1.0) for consideration of functional evaluations, as the disease-associated *SAMD9L* p.R986C (c.2956C>T) variant displays a population frequency of 0.000011 in the European population (Fig. S1). Of the nine variants we examined, six were absent from the gnomAD database (24), and in three cases, variants were confirmed de novo, whereas in two of the other cases the variants were confirmed as inherited. As the clinical penetrance and expressivity of *SAMD9* and *SAMD9L* variants are highly variable, it was difficult to predict functional consequences of specific variants.

**Table 1.** Genetic and clinical characteristics of patients

Patient	Gene	Variant (cDNA)	Variant (Protein)	Somatic variants	MAF	CADD	Sex	Age at onset	Presentation	Mode of inheritance	Function
P1	*SAMD9*	c.584A>G	p.H195R	​	6.20e-7	21.7	Male	2 wk	Chronic herpes infection	ND	NoF
P2	*SAMD9*	c.2809C>A	p.Q937K	​	0	15.0	Female	2.5 mo	Acute respiratory failure due to rhinovirus and high-load CMV viremia	*De novo*	GoF
P3	*SAMD9*	c.4658T>C	p.I1553T	​	9.61e-5	22.4	Female	7 years	Fever, rash, abdominal pain, diarrhea, and hyperinflammation	ND	NoF
P4	*SAMD9L*	c.2037T>A	p.F679L	​	1.24e-6	18.0	Male	4 years	Varicella encephalitis	Inherited	WT
P5	*SAMD9L*	c.2419G>A	p.E807K	c.4571G>A (p.R1524H); c.4571_4574del (p.R1524QfsX9)	0	24.2	Female	20 years	Ataxia and pancytopenia	ND	GoF
P6	*SAMD9L*	c.2649_2650del	p.C883X	​	0	33.0	Female	2 days	Recurrent fever with skin lesions from birth and during the neonatal period	De novo	GoF
P7	*SAMD9L*	c.3313T>A	p.W1105R	No evidence of reversion	0	3.8	Male	50 years	Ataxia	ND	NoF
P8	*SAMD9L*	c.3526G>A	p.E1176K	No evidence of reversion	0	21.3	Female	30 years	Ataxia	Inherited (AD)	GoF
P9	*SAMD9L*	c.3841_3843del	p.R1281del	c.2527C>T (p.R843W)>	0	11.3	Male	1 year	Thrombocytopenia, easy bruising, nosebleeds, petechiae, eczema, and monosomy 7	*De novo*	GoF

AD, autosomal dominant; ND, not determined; NoF, non-GoF; MAF, minor allele frequency.

### Functional characterization of patient-derived *SAMD9* and *SAMD9L* variants

**Figure 1.**
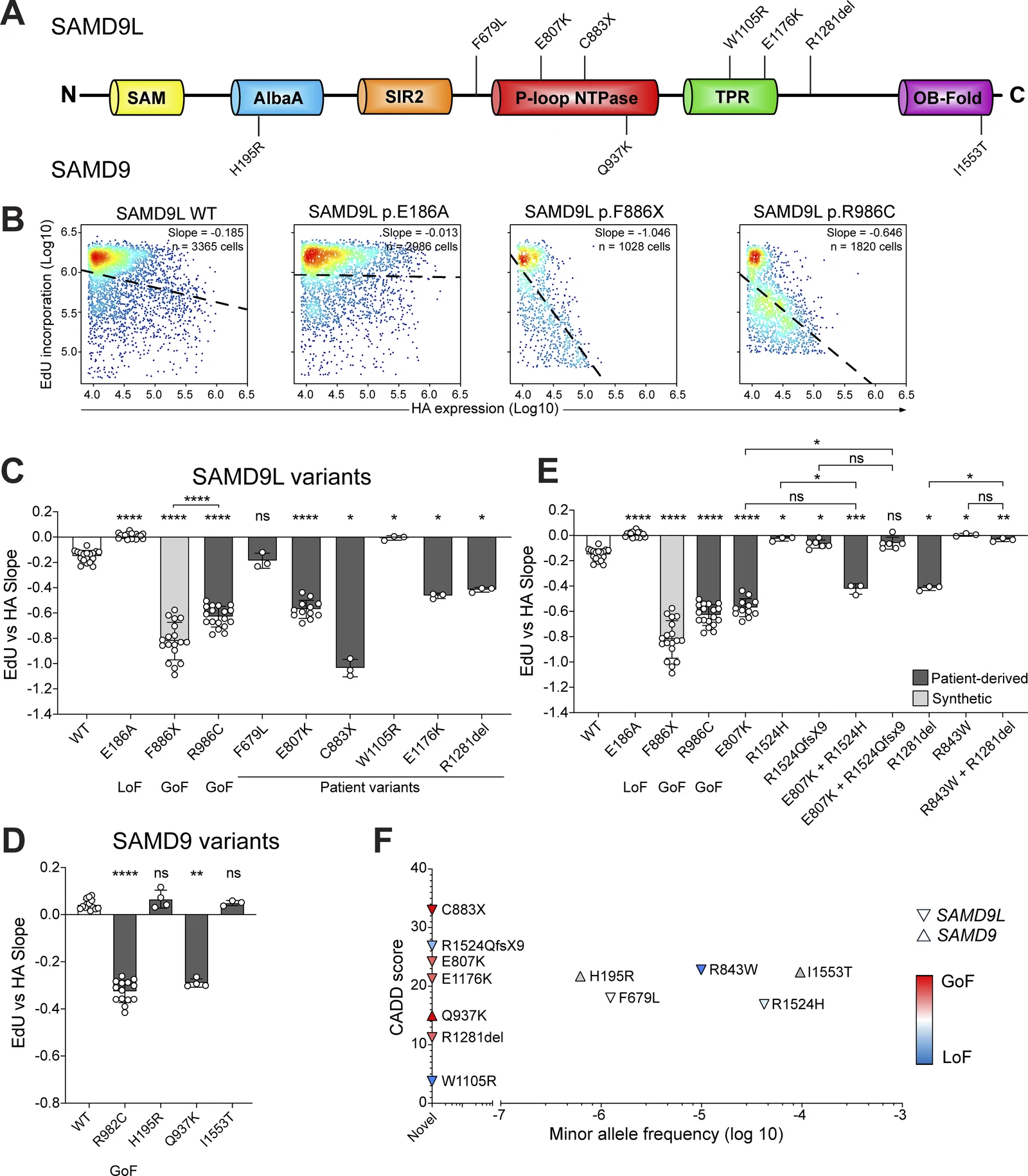
**Functional screening of various novel SAMD9 and SAMD9L patient variants. (A)** Schematic representation of SAMD9L and SAMD9 protein domain architecture, indicating the position of patient-derived variants. TPR, tetratricopeptide repeats; OB-fold, oligonucleotide/oligosaccharide-binding fold. **(B)** Representative scatter plots of EdU incorporation against HA signal intensity for four HA-tagged SAMD9L reference variants, illustrating the range of functional outcomes captured by the assay. The slope of the linear regression is indicated for each variant; increasingly negative slopes reflect progressively stronger inhibition of proliferation relative to HA-tagged SAMD9L protein expression. **(C)** EdU incorporation assay for HA-tagged SAMD9L germline patient variants, with previously characterized variants E186A (LoF), F886X (GoF), and R986C (GoF) included as controls. **(D)** EdU incorporation assay for SAMD9L somatic variants, with the parental germline variant included as reference where applicable. Compound variants represent single proteins carrying both mutations in cis. **(E)** EdU incorporation assay for SAMD9 patient variants, with R982C included as a GoF control. In panels C–E, dark grey bars indicate patient-derived variants and light grey bars indicate synthetic variants. Each data point represents an independent experiment, with a minimum of three replicates per variant. **(F)** CADD phred scores plotted against minor allele frequency for all patient-derived *SAMD9* and *SAMD9L* variants, color-coded by functional classification: GoF (red), LoF (blue). *P < 0.05, **P < 0.01, ***P < 0.001, ****P < 0.0001; ns, not significant.

**Figure S2.**
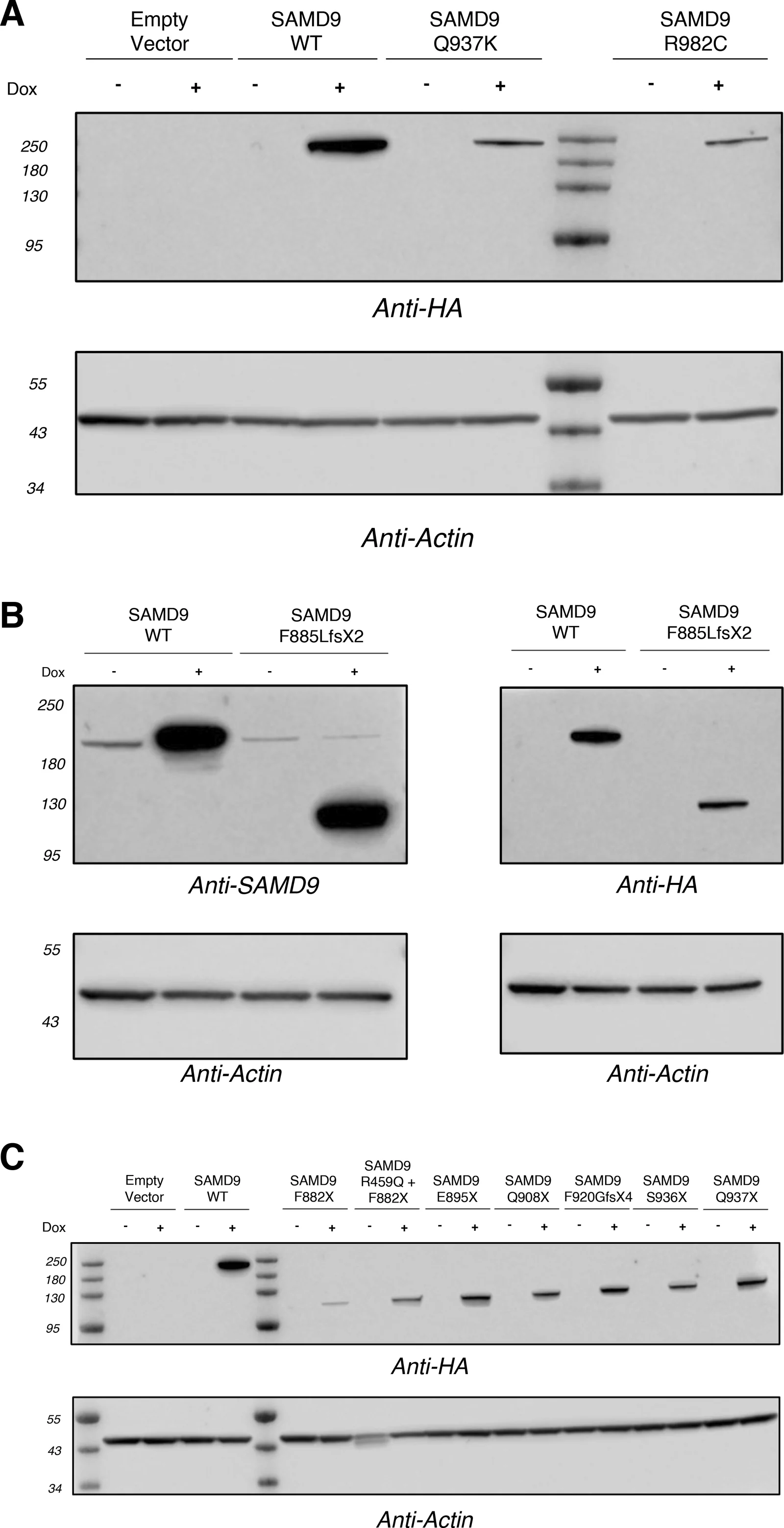
**Expression of SAMD9 variants in stable HEK 293T cell lines.** Immunoblot analysis of stable HEK 293T cell lines expressing doxycycline-inducible SAMD9 variants, with doxycycline-treated (+) and untreated (−) conditions shown for each line. **(A)** Expression of selected SAMD9 variants detected with anti-HA antibody. **(B)** Expression of SAMD9 p.F885LfsX2 detected with both anti-SAMD9 and anti-HA antibodies, with WT included for comparison. **(C)** Expression of SAMD9 truncating variants across the P-loop NTPase region detected with anti-HA antibody. β-actin was included as a loading control in all panels. Source data are available for this figure: SourceData FS2.

**Figure S3.**
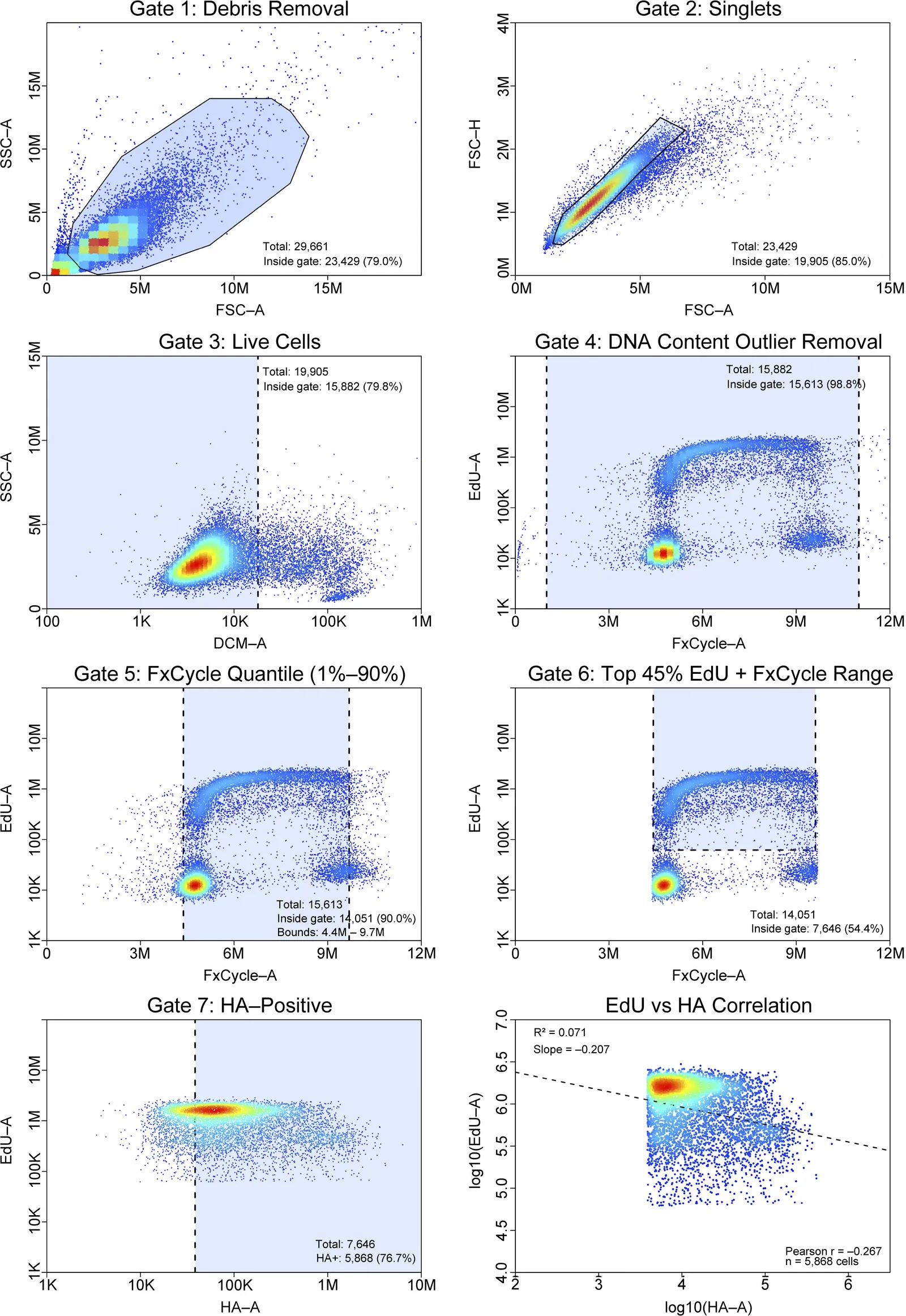
**EdU assay gating strategy and correlation approach.** Representative gating strategy for EdU incorporation analysis, shown for SAMD9L V1512M (Dox+). Gates 1–2: Debris exclusion (forward scatter area versus side scatter area [FSC-A vs. SSC-A]) and singlet selection (FSC-A vs. forward scatter height [FSC-H]). Gate 3: Live cell selection based on LIVE/DEAD Near-IR staining (dead cell marker [DCM-A]). Gate 4: Removal of S-phase outliers (top and bottom 0.1 percentile excluded). Gate 5: FxCycle quantile filtering, retaining cells between the 1st and 90th percentile of DNA content. Gate 6: Selection of the top 45% of EdU signal within the Gate 5 population. Gate 7: HA-positive cell selection, with the threshold defined as the 99th percentile of HA signal from doxycycline-untreated empty vector control cells for each experiment. The final panel shows the EdU vs. HA correlation for HA-positive cells; the slope of the linear regression was calculated for each variant per experiment and used as the metric for functional classification.

To functionally assess the patient-derived *SAMD9* and *SAMD9L* variants (Fig. 1 A), we generated stable HEK 293T cell lines expressing doxycycline-inducible, N-terminally 3×HA-tagged variants of each protein (Fig. S2, A and B). Given that upon induction of protein expression, GoF variants in SAMD9 and SAMD9L restrict cell proliferation by depleting tRNA^Phe^ and thereby blocking cell cycle progression (20), we employed a flow cytometry-based EdU incorporation assay to quantify cells in S-phase following 24 h of transgene induction. To account for differences in expression levels between variants and among cells, we calculated the slope of EdU incorporation against HA-SAMD9 or HA-SAMD9L signal intensity, with negative slopes indicating reduced proliferation relative to protein expression. Since standard flow cytometry analysis tools are not optimized for correlating proliferation with variable transgene expression levels, we developed a custom computational pipeline enabling automated, high-throughput quantification of EdU incorporation normalized to hemagglutinin (HA) signal intensity across thousands of cells per sample (Fig. 1 B and Fig. S3). As positive controls, we included the previously characterized *SAMD9L* p.F886X (c.2657_2658TT>GA), *SAMD9L* p.R986C (c.2956C>T), and *SAMD9* p.R982C (c.2944C>T) GoF variants, as well as *SAMD9L* p.E186A (c.557A>C) as an LoF control (20, 25, 26). Using this approach, we identified several novel GoF variants, including *SAMD9L* p.E807K, p.C883X, p.E1176K, and p.R1281del, as well as *SAMD9* p.Q937K (Fig. 1, C and D). Of these, *SAMD9L* p.C883X displayed the strongest GoF activity among the newly identified variants. Notably, all variants designated as GoF localized to the C-terminal region of their respective proteins (Fig. 1 A). *SAMD9L* p.F679L did not differ significantly from WT and likely represents a neutral variant (Fig. 1 C). We also note that for *SAMD9* p.H195R, p.I1553T, *SAMD9L* p.F679L, and p.W1105R, clinical whole-genome sequencing (WGS) did not yield an alternative molecular diagnosis or identify a strong alternative candidate variant.

In two of the patients, variants with reduced allele frequency were identified alongside their germline variants, prompting us to test whether these exhibited LoF activity characteristic of adaptive reversions (Fig. 1 E). *SAMD9L* p.R843W, identified as a somatic variant in the patient carrying *SAMD9L* p.R1281del, fully rescued the GoF phenotype when combined in cis. Similarly, two distinct somatic variants—*SAMD9L* p.R1524H and p.R1524QfsX9—were found in the patient with germline *SAMD9L* p.E807K. Of these, the *SAMD9L* p.R1524QfsX9 variant completely rescued the GoF activity of p.E807K, whereas the effect of p.R1524H appeared partial and was not statistically significant.

To assess to what degree Combined Annotation Dependent Depletion (CADD) scores (27) could predict functional classification in our variant set, we plotted CADD phred scores against minor allele frequency (24) for all patient-derived variants (Fig. 1 F). No correlation between CADD score and functional class was observed among the patient variants in this study (R^2^ = 0.028), with GoF and LoF variants scattered across the range of scores (Fig. 1 F), a pattern that extends to the broader landscape of nonsynonymous variation in both genes as catalogued in gnomAD (Fig. S1).

### *SAMD9L* p.C883X represents the shortest truncating variant conferring GoF activity

**Figure 2.**
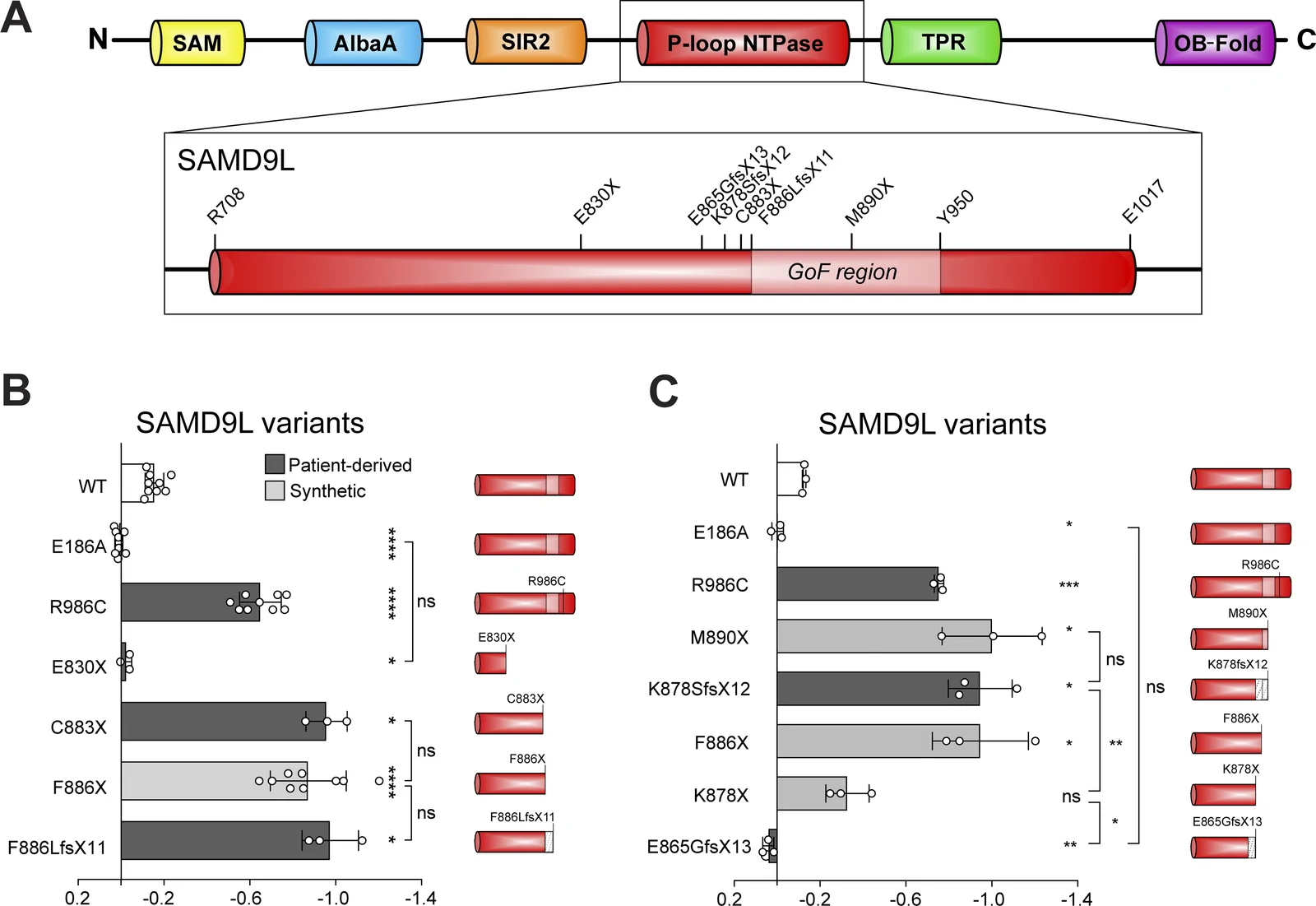
**Requirements for GoF activity in SAMD9L P-loop NTPase truncating variants. (A)** Schematic of SAMD9L protein domain architecture (top) with an expanded view of the P-loop NTPase domain (bottom). The highlighted region within the P-loop NTPase domain (C883–Y950) demarcates the boundaries where truncations yield a GoF phenotype. **(B and C)** EdU incorporation assay comparing C883X to previously reported SAMD9L truncations (B) and truncations within the P-loop NTPase domain, including variants with equivalent length but differing terminal sequences (C). Select data in B are reproduced from Fig. 1 B for comparison. Schematic representations of each truncated protein are shown to the right of panel C. Each data point represents an independent experiment, with a minimum of three replicates per variant. *P < 0.05, **P < 0.01, ***P < 0.001, and ****P < 0.0001; ns, not significant.

Truncating *SAMD9L* variants within its “GoF-conferring region,” previously proposed by Allenspach and colleagues to span F886–Y950(23) (Fig. 2 A), have been associated with severe, early-onset SAAD syndrome (5, 6). Among the novel variants identified in our patient cohort, *SAMD9L* p.C883X, lying N-terminally to this boundary, exhibited strong GoF activity in our assay, comparable with the established truncating variants *SAMD9L* p.F886X(23) and the patient-derived p.F886LfsX11 (25) (Fig. 2 B). To further investigate how far this boundary extends in the N-terminal direction, we tested p.E830X, previously reported (28) as the shortest SAMD9L truncating GoF variant, and found that it not only failed to display GoF activity but also displayed activity significantly lower than that of SAMD9L WT, exhibiting behavior more consistent with a LoF variant (Fig. 2 B). Similarly, other shorter truncations, including the synthetic p.K878X and the gnomAD p.E865GfsX13 *SAMD9L* variants, likewise failed to recapitulate GoF activity (Fig. 2, A and C). Notably, p.K878SfsX12, another truncating variant previously identified in patients as a GoF (4, 5), produces a protein that is in fact longer than p.C883X due to the frameshift-introduced residues.

To investigate the determinants of GoF activity within this region of *SAMD9L*, we generated a series of truncating variants differing in length and terminal sequence and compared their functional outcomes (Fig. 2 C). *SAMD9L* p.M890X and p.K878SfsX12 variants encode proteins of identical length that differ in their C-terminal amino acid sequences, yet both displayed GoF activity, suggesting that the specific sequence of the truncated C terminus does not impact GoF activity. Conversely, *SAMD9L* p.K878X and p.K878SfsX12 share the same initiating truncation site but differ in the resulting protein length—only the longer p.K878SfsX12, whose length exceeds 883 amino acids, exhibited GoF activity, while p.K878X behaved similarly to WT. These observations point to protein length, rather than terminal sequence, as the primary determinant of GoF activity within this region. However, the same relationship does not hold for more N-terminal truncations: the gnomAD p.E865GfsX13 and synthetic p.K878X variants both produce proteins shorter than p.C883X, yet their functional outcomes were not equivalent—p.E865GfsX13 showed no activity, while p.K878X exhibited residual activity resembling WT, suggesting that GoF potential may increase as truncation length approaches C883.

Taken together, these findings establish p.C883X as the shortest described *SAMD9L* truncation retaining GoF activity and extend the N-terminal boundary of the GoF-conferring region from ∼F886 to ∼C883. Furthermore, protein length rather than terminal sequence could determine GoF activity within the *SAMD9L* GoF-conferring region.

### Comparative analysis of P-loop NTPase truncating variants in *SAMD9* and *SAMD9L*

**Figure 3.**
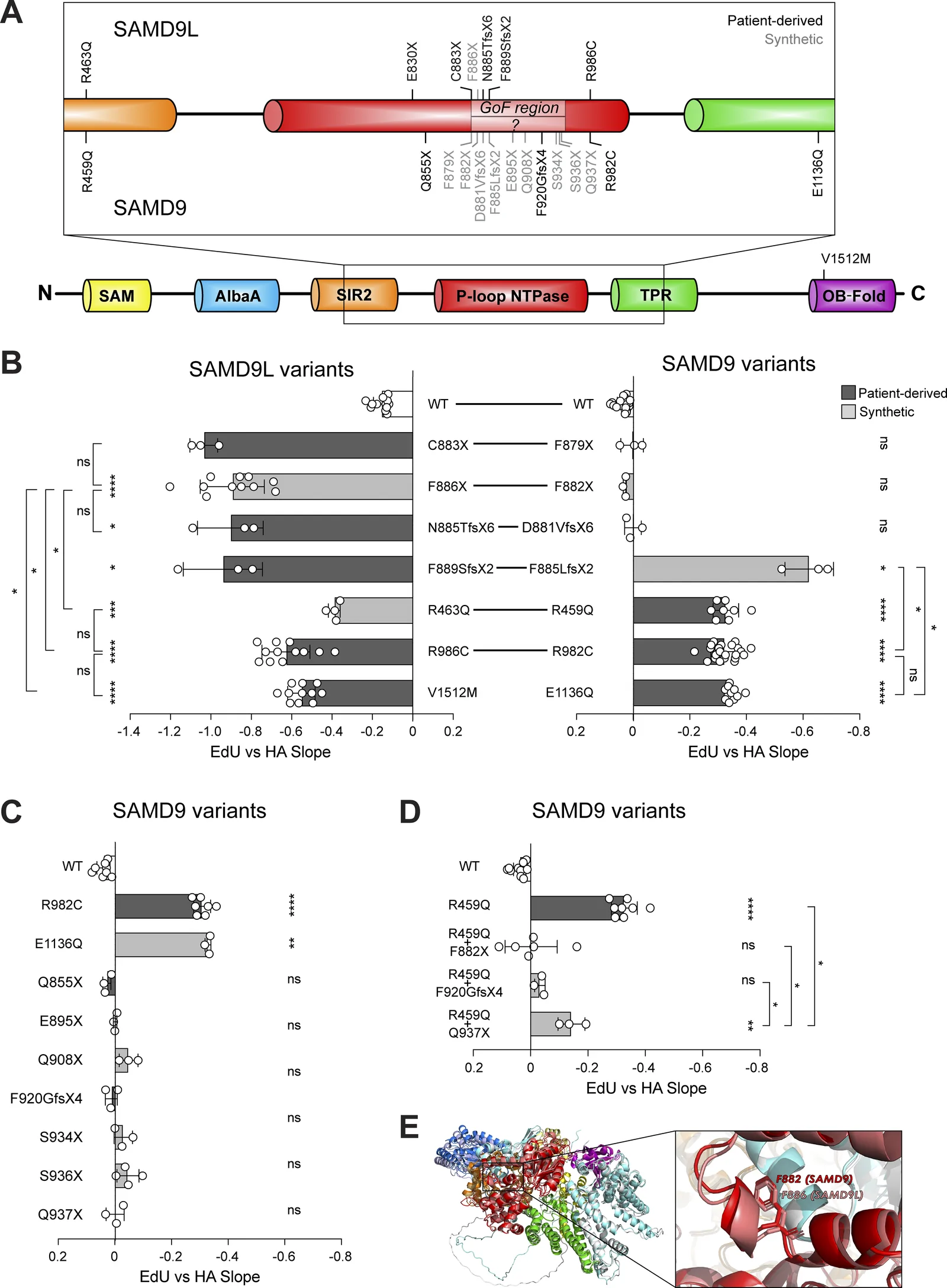
**Comparative analysis of P-loop NTPase truncating variants in SAMD9 and SAMD9L. (A)** Schematic of SAMD9L and SAMD9 protein domain architecture (bottom) with an expanded view of the P-loop NTPase domain (top), showing the position of truncating and missense variants. Corresponding homologous positions in SAMD9L and SAMD9 are aligned vertically. Black and grey text indicate patient-derived and synthetic variants, respectively. **(B)** EdU incorporation assay for SAMD9L variants at positions within and flanking the GoF-conferring window (left) and SAMD9 variants at homologous positions (right), with R464Q, R986C, and V1512M included as established SAMD9L GoF controls and R459Q, R982C, and E1136Q included as established SAMD9 GoF controls. Selected SAMD9L data are reproduced from Fig. 1 C for comparison. **(C)** EdU incorporation assay for SAMD9 truncations across the region homologous to the SAMD9L GoF-conferring window, with R982C and E1136Q included as GoF controls. **(D)** EdU incorporation assay for SAMD9 R459Q with and without truncating variants in cis. **(E)** Superimposition of AlphaFold2-predicted structures of SAMD9 (brighter colors) and SAMD9L (paler colors), with full-length proteins shown on the left and a magnified view of the F882 (SAMD9) and F886 (SAMD9L) region on the right. Each data point in B–D represents an independent experiment, with a minimum of three replicates per variant. *P < 0.05, **P < 0.01, ***P < 0.001, ****P < 0.0001; ns, not significant.

Having refined the region of the *SAMD9L* P-loop NTPase domain where truncating variants consistently display GoF activity, we next examined whether analogous truncations in *SAMD9* behave similarly (Fig. 3 A). While no truncating *SAMD9L* variants within this region appear in gnomAD, several *SAMD9* truncations (p.E895X, p.S934X, p.S936X, and p.Q937X) are present at minor allele frequencies as high as 1.3 × 10^–5^. Additionally, a truncating *SAMD9* frameshift variant in this region, p.F920GfsX4, has also been reported as a somatic revertant (28) (Fig. 3 A), suggesting distinct regulation of activity between *SAMD9* and *SAMD9L*. In *SAMD9L*, truncating variants associated with SAAD exhibited stronger GoF activity than germline missense variants associated with ATXPC (Fig. 3 B, left). To test whether this pattern holds for *SAMD9*, we generated truncations at positions homologous to those in *SAMD9L*. Strikingly, SAMD9 truncations at these positions consistently failed to produce a GoF phenotype (Fig. 3 B, right). The sole exception, *SAMD9* p.F885LfsX2, not only retained GoF activity but exhibited stronger activity than the established *SAMD9* GoF missense variants associated with MIRAGE syndrome: p.R459Q (29), p.R982C (30), and p.E1136Q (31). To further explore this discrepancy, we performed a mutational scan across the corresponding region in *SAMD9* (Fig. 3 C). None of the truncations tested yielded a GoF phenotype, reinforcing the notion that p.F885LfsX2 represents a rare exception. To determine whether these *SAMD9* truncations confer a LoF phenotype, we combined some of them in cis with the GoF variant p.R459Q (Fig. 3 D). Each truncation was able to rescue the GoF phenotype, confirming LoF activity. Of note, the shorter p.F882X fully rescued p.R459Q, while the longer p.R459Q + p.Q937X compound variant retained partial activity, suggesting that rescue capacity inversely correlates with the length of the resulting peptide. Western blot analysis confirmed that all truncated SAMD9 variants were expressed at their expected molecular weights, excluding protein instability as an explanation for their LoF phenotype (Fig. S2 C).

Comparison of AlphaFold2-predicted (32) structures of SAMD9 and SAMD9L revealed near-identical conformations, both overall and particularly in the region surrounding the F882 (SAMD9) and F886 (SAMD9L) residues (Fig. 3 E), a region where truncations yield LoF activity in SAMD9 but GoF activity in SAMD9L.

Taken together, these results suggest stronger inhibition of cell cycle by de novo* SAMD9L* truncating variants associated with SAAD compared with missense *SAMD9L* variants associated with ATXPC. Strikingly, SAMD9 truncations at homologous positions generally failed to yield a GoF phenotype, instead exhibiting length-dependent LoF activity—revealing an unanticipated functional divergence between the two paralogues.

### Established computational pathogenicity predictors do not reliably distinguish GoF from LoF variants in *SAMD9* and *SAMD9L*

**Figure 4.**
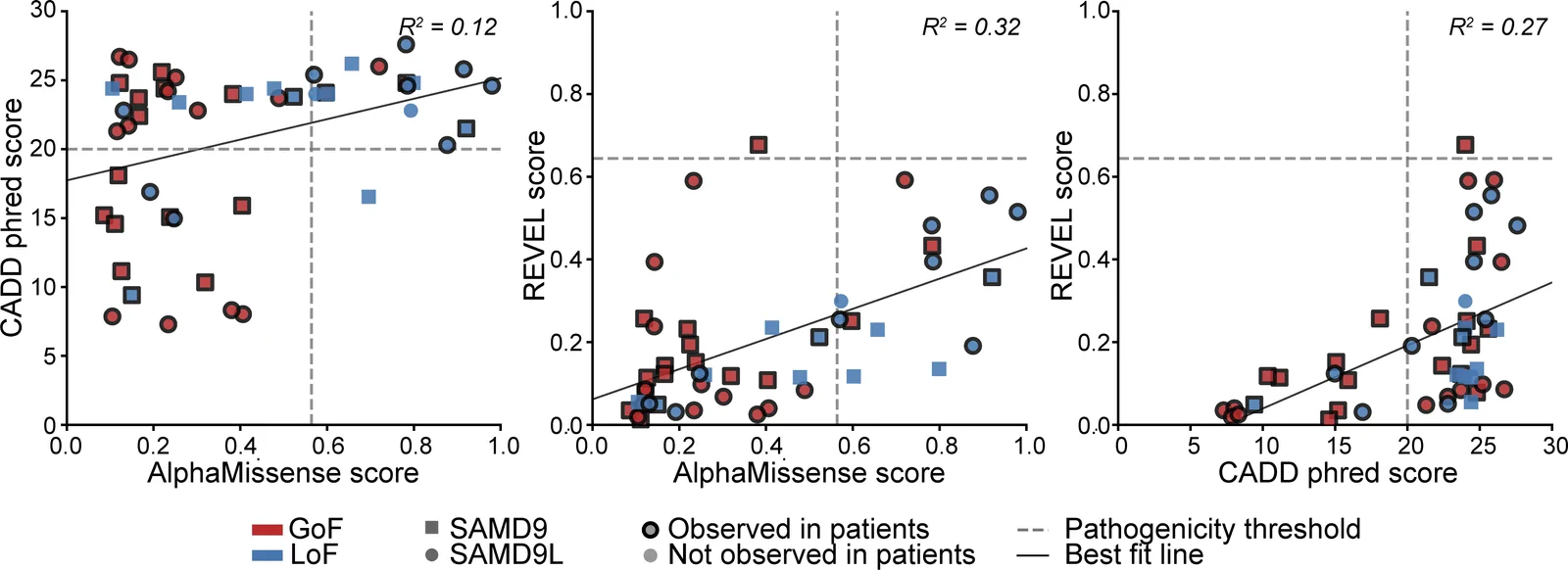
**Computational pathogenicity prediction tools are mutually discordant and fail to reliably classify known pathogenic SAMD9 and SAMD9L missense variants.** Pairwise scatter plots of AlphaMissense, CADD, and REVEL scores for a curated set of 50 *SAMD9* (squares) and *SAMD9L* (circles) missense variants collated from published functional studies. Red, GoF; blue, LoF. Variants observed in patients are outlined in black. Dashed lines indicate established pathogenicity thresholds (AlphaMissense ≥0.564; CADD phred ≥20; REVEL ≥0.644). The solid black line represents the linear best fit; R^2^ values are shown. Two LoF variants (*SAMD9* p.Arg221Glu and *SAMD9L* p.Arg223Glu) are omitted from panels containing REVEL scores due to their dinucleotide nature, which precludes REVEL scoring.

To evaluate the utility of computational pathogenicity prediction tools in classifying *SAMD9* and *SAMD9L* variants by functional category, we collated CADD (33), Rare Exome Variant Ensemble Learner (REVEL) (34), and AlphaMissense (35) scores for a curated set of 50 *SAMD9* and *SAMD9L* missense variants with established functional annotations drawn from published studies, including variants characterized in the present work (Fig. 4 and Table S2). Pairwise comparison of the three scores revealed poor mutual agreement, with R^2^ values of 0.32 between AlphaMissense and REVEL, 0.27 between CADD and REVEL, and 0.12 between AlphaMissense and CADD, indicating that the tools provide substantially discordant assessments of the same variants. More importantly, when variants were stratified by functional class, GoF and LoF variants were broadly intermingled across all three scoring systems, with neither category occupying a distinct region of score space. Since both GoF and LoF variants are pathogenic—albeit through divergent mechanisms—existing pathogenicity thresholds do not resolve this ambiguity: every variant in the dataset, regardless of functional class, scored below previously employed cutoffs for at least one tool (CADD phred <20 [36], REVEL <0.644 [37], or AlphaMissense <0.564 [38]), and would therefore be misclassified as benign in a purely computational assessment.

## Discussion

*SAMD9* and *SAMD9L* variants are associated with a remarkably broad spectrum of clinical presentations, ranging from myeloid malignancies and bone marrow failure to autoinflammatory diseases, inborn errors of immunity, and neurological disorders, including ataxia. This phenotypic diversity, combined with variable penetrance and expressivity, makes clinical interpretation of novel variants particularly challenging. Here, we address this challenge through systematic functional characterization of patient-derived variants, yielding three principal findings. First, the N-terminal boundary of the *SAMD9L* GoF-conferring region is revised from ∼F886 to ∼C883, with protein length rather than terminal sequence emerging as the primary determinant of GoF activity within this window. Second, *SAMD9* truncations at positions homologous to *SAMD9L* GoF variants consistently yield LoF rather than GoF phenotypes, revealing an unanticipated functional divergence between these structurally similar paralogues. Third, established computational pathogenicity predictors fail to reliably identify pathogenic variants or distinguish GoF from LoF variants in this gene family, underscoring that empirical functional testing remains indispensable for accurate variant interpretation.

The interpretive challenge is further compounded by the cellular toxicity of GoF variants, which drives strong selection for somatic reversion: secondary LoF mutations, uniparental disomy, or chromosomal loss can all attenuate the pathogenic allele and, in some cases, lead to spontaneous remission of hematological symptoms (8). *SAMD9* and *SAMD9L* have also evolved under complex and at times conflicting selective pressures—including purifying selection, pathogen-driven positive selection, and strong somatic counter-selection against GoF alleles (17, 39, 40)—a background that may confound tools such as CADD (33), REVEL (34), and AlphaMissense (35), which are calibrated on the assumption of predominantly purifying selection. These challenges highlight a broader principle: in rapidly evolving gene families with complex selective histories (39), variant pathogenicity cannot be assumed from population genetics or computational inference alone.

Our findings also redefine the boundaries of the GoF-conferring region within the *SAMD9L* P-loop NTPase domain. The identification of p.C883X as a GoF variant extends the N-terminal boundary from the previously defined ∼F886 to at least ∼C883. This also addresses a conflicting report in the literature: p.E830X, reported as a GoF variant by Sahoo and colleagues (28), lies N-terminally to this revised boundary, and in our assay exhibited behavior consistent with LoF rather than GoF activity—a discrepancy that may reflect differences in experimental systems. Consistent with these boundaries, the only truncating variant within this region present in gnomAD, p.Y931MfsX30, produces a protein of 961 amino acids—a length that exceeds the established C-terminal boundary of the GoF-conferring region (∼Y950), which would explain its presence in a database of ostensibly healthy individuals. Within this region, protein length rather than terminal sequence identity determines GoF activity—a finding that argues against a model in which specific C-terminal residues are required for GoF activity and instead supports removal of an autoinhibitory domain as the underlying mechanism. Consistent with this model, truncating variants exhibited stronger GoF activity than missense variants at comparable positions, suggesting that missense variants may retain partial autoinhibitory function. We also observed that direct nonsense mutations (e.g., *SAMD9L* p.F886X, p.C883X) and frameshift variants that introduce additional residues before a premature stop codon (e.g., *SAMD9L* p.F886LfsX11, p.F889SfsX2) behave similarly, indicating that the addition of novel amino acids does not alter the functional outcome so long as the resulting protein length remains within the GoF-conferring range.

The most unexpected finding of this study, however, is the divergent behavior of analogous truncations in *SAMD9* vs. *SAMD9L*. Despite high sequence identity and near-identical predicted structures in the region surrounding the truncation sites, *SAMD9L* truncations consistently yield GoF phenotypes, while homologous *SAMD9* truncations typically produce LoF. The sole exception, *SAMD9* p.F885LfsX2, exhibited potent GoF activity, yet truncations just a few residues away functioned as LoF variants capable of rescuing GoF activity. The mechanistic basis for this divergence remains unclear. Subtle differences in protein dynamics, interaction partners, or posttranslational modifications—not captured in static structural predictions—may underlie these distinct outcomes. The ∼40% amino acid sequence divergence between the two paralogues, concentrated in regions outside the structurally conserved core, may be sufficient to alter the allosteric consequences of truncation even when the local folding appears equivalent. The recently determined cryo-EM structures of both SAMD9 and SAMD9L, which reveal the structural basis of autoinhibition and its disruption by GoF mutations, provide an important framework for future investigations into this divergence (41, *Preprint*, 42, *Preprint*). It is tempting to speculate, however, that this divergence might reflect the distinct expression profiles of the two paralogues: in many cell types SAMD9 is expressed constitutively at relatively higher baseline levels, whereas SAMD9L expression is more strongly induced by interferon signaling (43). A constitutively expressed restriction factor may therefore require tighter autoinhibitory control to prevent promiscuous activation, and this constraint may render SAMD9 more sensitive to the destabilizing consequences of truncation, converting what would otherwise be a GoF event in SAMD9L into a LoF outcome in SAMD9. Under this model, the paralogue-specific divergence in truncation outcomes would reflect not a difference in local structure at the truncation site, but an evolved difference in the regulatory profile of the two proteins—a distinction that static structural comparisons alone would not be expected to reveal.

Beyond the challenge of variant classification, we also observed that LoF variants exist on a spectrum: some, such as *SAMD9L* p.R843W, completely abolished GoF activity when combined in cis, while others, such as *SAMD9L* p.R1524H, only partially attenuated it. This spectrum of partial LoF activity has not been widely appreciated in the *SAMD9/SAMD9L* field, where variants are often treated as binary. The degree of residual activity may have clinical relevance, potentially influencing disease severity or the selective pressure for somatic reversion. In addition, although de novo GoF variants might a priori be expected to exhibit stronger activity than inherited ones, our, albeit limited, data do not support a clear trend in this regard.

As clinical genome sequencing increasingly identifies rare *SAMD9* and *SAMD9L* variants of uncertain significance, these findings argue that empirical functional characterization should be considered an integral component of the diagnostic workup in this gene family, in line with recent discussion regarding the American College of Medical Genetics and Genomics’ recommendations for interpretation of sequence variants (44).

## Materials and methods

### Patients and sequencing

WGS aimed at finding germline variants was performed on DNA extracted from whole blood as described in (45). Sequencing as well as bioinformatic analysis was performed at the Clinical Genomics facility at SciLifeLab as part of the clinical collaboration Genomic Medicine Center Karolinska Rare Disease. The bioinformatic pipeline “MIP” was used for all samples but has been developed over the years; details are available at: https://github.com/Clinical-Genomics/MIP. In silico panels for inborn errors of immunity or bone marrow failure were used to filter variants, depending on the clinical phenotype. Segregation of identified variants in parents was performed using Sanger sequencing on DNA extracted from whole blood. In some cases, trio analysis with WGS was performed directly, in which case confirmation analysis using Sanger was not necessary.

### Plasmid construction and site-directed mutagenesis

Primers for variants of interest were designed using the NEBaseChanger tool (https://nebasechanger.neb.com/). Novel variants were introduced into pENTR4_3XHA *SAMD9/SAMD9L* plasmids by site-directed mutagenesis using PCR amplification with Q5 Hot Start High-Fidelity 2X Master Mix (Cat# M0494S; NEB) and KLD Enzyme Mix (Cat# M0554S; NEB) according to the manufacturer’s instructions. Mutated *SAMD9/SAMD9L* genes were subsequently transferred into the PB-TA-ERP2 expression vector (Gateway LR Clonase II Enzyme Mix, Cat# 11791020; Thermo Fisher Scientific). Resulting plasmids were verified by a combination of Sanger sequencing and nanopore-based whole-plasmid sequencing.

### Cell culture

HEK 293T cells (ATCC) were cultured in high glucose DMEM with sodium pyruvate (DMEM + GlutaMAX, Cat# 31966021; Gibco) supplemented 10% FBS. The cells were kept at 37°C with 5% CO_2_.

### Cell line generation/Transfections

HEK 293T cells (2.5 × 10^5^) were seeded in 6-well plates with DMEM + GlutaMAX supplemented with 10% FBS and cultured overnight. Cells were transfected with 2 μg of PB-TA-ERP2 expression vector encoding the SAMD9/SAMD9L variant of interest and 0.5 μg of the Super PiggyBac Transposase vector (Cat# V012800; NovoPro). Plasmid DNA was diluted in 250 μl Opti-MEM reduced serum medium and transfected with 7.5 μl of TransIT-LT1 reagent (Cat# 2304; Mirus Bio) according to the manufacturer’s instructions. Transfected cells were selected with puromycin (1 μg/ml) for 6 days to establish stable cell lines.

### Flow cytometry and EdU assays

Cells were seeded at 1 × 10^4^ cells per well in 96-well plates in triplicate and allowed to adhere overnight. After 24 h, cells were treated with doxycycline (1 μg/ml) for 24 h to induce variant expression, then incubated with 10 μM EdU (Cat# CLK-N001-100; Jena Bioscience) for 1 h at 37°C. Cells were then detached by vigorous pipetting, washed with FACS buffer (Dulbecco’s phosphate-buffered saline [DPBS] with 2% FCS and 2 mM EDTA), and pelleted by centrifugation at 400 × *g* for 5 min. Cells were stained with LIVE/DEAD Fixable Near-IR Dead Cell Stain Kit (Cat# L34975; Thermo Fisher Scientific; 1:1,000) for 5 min at room temperature, washed with FACS buffer, fixed with 2% paraformaldehyde in PBS for 12 min at 37°C, permeabilized with 0.01% Triton X-100 for 7 min at room temperature, and washed again. For EdU detection, cells were incubated for 30 min at room temperature with a click reaction mixture containing 4 mM CuSO_4_, 10 mM ascorbic acid, and 1 μM Alexa Fluor 488 Azide (Cat# A10266; Thermo Fisher Scientific). Cells were then washed and incubated for 30 min at room temperature with APC anti-HA.11 Epitope Tag antibody (Cat# 901524; BioLegend; 1:100) and either anti-SAMD9 (Cat# HPA021319; Atlas Antibodies; 1:400) or anti-SAMD9L (Cat# HPA019465; Atlas Antibodies; 1:200), followed by washing and incubation with goat anti-rabbit IgG Alexa Fluor 546 secondary antibody (Cat# A-11035; Thermo Fisher Scientific; 1:1,000) for 15 min at room temperature. After washing, cells were stained with FxCycle Violet Ready Flow Reagent (Cat# R37166; Thermo Fisher Scientific) for 30 min at room temperature and washed again. Samples were analyzed using a CytoFLEX Flow Cytometer (Beckman Coulter), and data were processed using a custom pipeline (https://github.com/pmitev93/Flow_EdU.git).

### Western blotting

Cells were seeded at 2.5 × 10^5^ cells per well in 6-well plates and allowed to adhere overnight. After 24 h, cells were treated with doxycycline (1 μg/ml) for 24 h. Cells were then detached by vigorous pipetting, washed with PBS, and lysed in ice-cold radioimmunoprecipitation assay buffer (50 mM Tris-HCl [pH 8.0], 150 mM NaCl, 0.5% NP-40, 2 mM EDTA; Cat# sc-24948; Santa Cruz Biotechnology) for 40 min on ice. Protein concentration was determined using the Pierce BCA Protein Assay Kit (Cat# 23225; Thermo Fisher Scientific) according to the manufacturer’s instructions. Samples were heat-denatured at 80°C for 10 min in 4X LDS Sample Buffer (Cat# NP0008; Invitrogen) and separated by SDS-PAGE using NuPAGE 4-12% Bis-Tris Midi Protein Gels (Cat# NP0335BOX; Invitrogen) in an XCell SureLock Mini-Cell system (Invitrogen). Proteins were transferred to polyvinylidene difluoride (PVDF) membranes using the iBlot 2 Transfer System with iBlot 2 PVDF Transfer Stacks (Cat# IB24002; Invitrogen). Membranes were blocked in TBS with 0.2% Tween-20 (TBS-T) containing 5% nonfat dry milk (Cat# 1153630500; Merck) for 1 h at room temperature. Membranes were then incubated with primary antibodies diluted in TBS-T overnight at 4°C, washed, and then incubated with HRP-conjugated secondary antibodies diluted in TBS-T for 1 h at room temperature. Protein bands were visualized by detection of HRP activity using SuperSignal West Dura Extended Duration Substrate (Cat# 34076; Thermo Fisher Scientific) on an Odyssey Fc Imaging System (LI-COR).

### Variant annotation

CADD scores were obtained using CADD v1.7 (https://cadd.gs.washington.edu/; GRCh38). REVEL scores were retrieved from the precomputed genome-wide dataset (v1.3; Zenodo: https://zenodo.org/records/7072866), with lookups restricted to chromosome 7 variants. AlphaMissense pathogenicity scores were obtained via the AlphaMissense web interface (https://alphamissense.hegelab.org/). Variant allele frequencies were retrieved from the Genome Aggregation Database (gnomAD v4.1.0; https://gnomad.broadinstitute.org/).

### Structural analysis

Structural analysis was performed on the AlphaFold2-generated models of SAMD9 (AF-Q5K651-F1) and SAMD9L (AF-Q8IVG5-F1) using PyMOL.

### Data visualization and statistical analysis

Data visualization and statistical analysis were performed using GraphPad Prism 10 software (GraphPad Software). For experiments comparing multiple variants, repeated-measures one-way ANOVA was used with either Dunnett’s post hoc test (for comparisons with control) or Šidák’s/Holm–Šidák’s multiple comparisons test (for pairwise comparisons between variants). Data were assumed to follow a Gaussian distribution. Geisser-Greenhouse correction was applied to account for violations of sphericity where applicable. Statistical significance was defined as P < 0.05. Data are presented as mean ± SD from at least three independent experiments.

### Online supplemental material

Fig. S1 shows CADD phred scores plotted against minor allele frequency for all nonsynonymous variants in *SAMD9* and *SAMD9L* catalogued in gnomAD, with confirmed disease-associated variants annotated. Fig. S2 shows western blot analysis of doxycycline-dependent protein expression for selected *SAMD9* and *SAMD9L* variants, including anti-HA, anti-SAMD9, and anti–β-actin detection. Fig. S3 describes the gating strategy and slope calculation approach used for the EdU incorporation assay. Table S1 provides extended clinical phenotypes of the patients described in this study. Table S2 lists the CADD, REVEL, and AlphaMissense scores alongside functional classifications for the 50 SAMD9 and SAMD9L missense variants analyzed in Fig. 4.

## Ethics statement

### Ethics approval

The study was approved by the Regional Ethical Review Boards in Stockholm and the Swedish Ethical Review Authority. Patients and relatives were recruited with informed written consent.

## Supplementary Material

Table S1 shows extended clinical phenotype of patients.

Table S2 shows computational pathogenicity scores and clinical annotations for *SAMD9* and *SAMD9L* variants.

SourceData FS2 is the source file for Fig. S2.

## Data Availability

To protect patient confidentiality in accordance with the European Union General Data Protection Regulation (GDPR) and Swedish data protection laws, sequencing data and other information are available via controlled access according to national GDPR rules upon request to the corresponding author. All other data are available upon reasonable request.

## References

[1] Sahoo, S.S., M. Erlacher, and M.W. Wlodarski. 2025. Genetic and clinical spectrum of SAMD9 and SAMD9L syndromes: From variant interpretation to patient management. Blood. 145:475–485. 10.1182/blood.2022017717 PMC11826520 39475954

[2] Narumi, S., N. Amano, T. Ishii, N. Katsumata, K. Muroya, M. Adachi, K. Toyoshima, Y. Tanaka, R. Fukuzawa, K. Miyako, . 2016. SAMD9 mutations cause a novel multisystem disorder, MIRAGE syndrome, and are associated with loss of chromosome 7. Nat. Genet. 48:792–797. 10.1038/NG.3569 27182967

[3] Suntharalingham, J.P., M. Ishida, I. Del Valle, S.E. Stalman, N. Solanky, E. Wakeling, G.E. Moore, J.C. Achermann, and F. Buonocore. 2022. Emerging phenotypes linked to variants in SAMD9 and MIRAGE syndrome. Front. Endocrinol. (Lausanne). 13:953707. 10.3389/FENDO.2022.953707/BIBTEX PMC9433874 36060959

[4] de Jesus, A.A., Y. Hou, S. Brooks, L. Malle, A. Biancotto, Y. Huang, K.R. Calvo, B. Marrero, S. Moir, A.J. Oler, . 2020. Distinct interferon signatures and cytokine patterns define additional systemic autoinflammatory diseases. J. Clin. Invest. 130:1669–1682. 10.1172/JCI129301 PMC7108905 31874111

[5] Russell, A.J., P.E. Gray, J.B. Ziegler, Y.J. Kim, S. Smith, W.A. Sewell, and C.C. Goodnow. 2021. SAMD9L autoinflammatory or ataxia pancytopenia disease mutations activate cell-autonomous translational repression. Proc. Natl. Acad. Sci. USA. 118:e2110190118. 10.1073/PNAS.2110190118 PMC8403910 34417303

[6] Papa, R., M. Rusmini, S. Volpi, G. Dell’Orso, M.C. Giarratana, R. Caorsi, S. Giardino, P. Bocca, P. Barone, M. Severino, . 2022. Progression of non-hematologic manifestations in SAMD9L-associated autoinflammatory disease (SAAD) after hematopoietic stem cell transplantation. Pediatr. Allergy Immunol. 33:e13711. 10.1111/PAI.13711 34894360

[7] Chen, D.-H., J.E. Below, A. Shimamura, S.B. Keel, M. Matsushita, J. Wolff, Y. Sul, E. Bonkowski, M. Castella, T. Taniguchi, . 2016. Ataxia-pancytopenia syndrome is caused by missense mutations in SAMD9L. Am. J. Hum. Genet. 98:1146–1158. 10.1016/J.AJHG.2016.04.009 PMC4908176 27259050

[8] Tesi, B., J. Davidsson, M. Voss, E. Rahikkala, T.D. Holmes, S.C.C. Chiang, J. Komulainen-Ebrahim, S. Gorcenco, A. Rundberg Nilsson, T. Ripperger, . 2017. Gain-of-function *SAMD9L* mutations cause a syndrome of cytopenia, immunodeficiency, MDS, and neurological symptoms. Blood. 129:2266–2279. 10.1182/BLOOD-2016-10-743302 PMC5399482 28202457

[9] Topaz, O., M. Indelman, I. Chefetz, D. Geiger, A. Metzker, Y. Altschuler, M. Choder, D. Bercovich, J. Uitto, R. Bergman, . 2006. A deleterious mutation in SAMD9 causes normophosphatemic familial tumoral calcinosis. Am. J. Hum. Genet. 79:759–764. 10.1086/508069 PMC1592555 16960814

[10] Hershkovitz, D., Y. Gross, S. Nahum, S. Yehezkel, O. Sarig, J. Uitto, and E. Sprecher. 2011. Functional characterization of SAMD9, a protein deficient in normophosphatemic familial tumoral calcinosis. J. Invest. Dermatol. 131:662–669. 10.1038/jid.2010.387 PMC3169306 21160498

[11] Chefetz, I., D. Ben Amitai, S. Browning, K. Skorecki, N. Adir, M.G. Thomas, L. Kogleck, O. Topaz, M. Indelman, J. Uitto, . 2008. Normophosphatemic familial tumoral calcinosis is caused by deleterious mutations in SAMD9, encoding a TNF-alpha responsive protein. J. Invest. Dermatol. 128:1423–1429. 10.1038/SJ.JID.5701203 PMC3169318 18094730

[12] Molteni, E., E. Bono, A. Gallì, C. Elena, J. Ferrari, N. Fiorelli, S. Pozzi, V.V. Ferretti, M. Sarchi, E. Rizzo, . 2023. Prevalence and clinical expression of germ line predisposition to myeloid neoplasms in adults with marrow hypocellularity. Blood. 142:643–657. 10.1182/BLOOD.2022019304 PMC10644067 37216690

[13] Nagamachi, A., H. Matsui, H. Asou, Y. Ozaki, D. Aki, A. Kanai, K. Takubo, T. Suda, T. Nakamura, L. Wolff, . 2013. Haploinsufficiency of SAMD9L, an endosome fusion facilitator, causes myeloid malignancies in mice mimicking human diseases with monosomy 7. Cancer Cell. 24:305–317. 10.1016/J.CCR.2013.08.011 24029230

[14] Cammenga, J. 2024. Of gains and losses: SAMD9/SAMD9L and monosomy 7 in myelodysplastic syndrome. Exp. Hematol. 134:104217. 10.1016/J.EXPHEM.2024.104217 38649131

[15] Davidsson, J., A. Puschmann, U. Tedgård, D. Bryder, L. Nilsson, and J. Cammenga. 2018. SAMD9 and SAMD9L in inherited predisposition to ataxia, pancytopenia, and myeloid malignancies. Leukemia. 32:1106–1115. 10.1038/S41375-018-0074-4 PMC5940635 29535429

[16] Schwartz, J.R., J. Ma, T. Lamprecht, M. Walsh, S. Wang, V. Bryant, G. Song, G. Wu, J. Easton, C. Kesserwan, . 2017. The genomic landscape of pediatric myelodysplastic syndromes. Nat. Commun. 8:1557. 10.1038/S41467-017-01590-5 PMC5691144 29146900

[17] Mekhedov, S.L., K.S. Makarova, and E.V. Koonin. 2017. The complex domain architecture of SAMD9 family proteins, predicted STAND-like NTPases, suggests new links to inflammation and apoptosis. Biol. Direct. 12:13. 10.1186/S13062-017-0185-2 PMC5445408 28545555

[18] Lemos de Matos, A., J. Liu, G. McFadden, and P.J. Esteves. 2013. Evolution and divergence of the mammalian SAMD9/SAMD9L gene family. BMC Evol. Biol. 13:121. 10.1186/1471-2148-13-121 PMC3685527 23758988

[19] Li, C.F., J.R. MacDonald, R.Y. Wei, J. Ray, K. Lau, C. Kandel, R. Koffman, S. Bell, S.W. Scherer, and B.A. Alman. 2007. Human sterile alpha motif domain 9, a novel gene identified as down-regulated in aggressive fibromatosis, is absent in the mouse. BMC Genomics. 8:92. 10.1186/1471-2164-8-92 PMC1855325 17407603

[20] Zhang, F., Q. Ji, J. Chaturvedi, M. Morales, Y. Mao, X. Meng, L. Dong, J. Deng, S.-B. Qian, and Y. Xiang. 2023. Human SAMD9 is a poxvirus-activatable anticodon nuclease inhibiting codon-specific protein synthesis. Sci. Adv. 9:eadh8502. 10.1126/SCIADV.ADH8502 PMC10246899 37285440

[21] Wong, J.C., V. Bryant, T. Lamprecht, J. Ma, M. Walsh, J. Schwartz, M. del pilar Alzamora, C.G. Mullighan, M.L. Loh, R. Ribeiro, . 2018. Germline SAMD9 and SAMD9L mutations are associated with extensive genetic evolution and diverse hematologic outcomes. JCI Insight. 3:e121086. 10.1172/JCI.INSIGHT.121086 PMC6124395 30046003

[22] Bluteau, O., M. Sebert, T. Leblanc, R.P. De Latour, S. Quentin, E. Lainey, L. Hernandez, J.-H. Dalle, F. Sicre de Fontbrune, E. Lengline, . 2018. A landscape of germ line mutations in a cohort of inherited bone marrow failure patients. Blood. 131:717–732. 10.1182/BLOOD-2017-09-806489 29146883

[23] Allenspach, E.J., F. Soveg, L.S. Finn, L. So, J.A. Gorman, A.B.I. Rosen, S. Skoda-Smith, M.M. Wheeler, K.A. Barrow, L.M. Rich, . 2021. Germline SAMD9L truncation variants trigger global translational repression. J. Exp. Med. 218:e20201195. 10.1084/JEM.20201195 PMC7970252 33724365

[24] Chen, S., L.C. Francioli, J.K. Goodrich, R.L. Collins, M. Kanai, Q. Wang, J. Alföldi, N.A. Watts, C. Vittal, L.D. Gauthier, . 2024. A genomic mutational constraint map using variation in 76,156 human genomes. Nature. 625:92–100. 10.1038/S41586-023-06045-0 PMC11629659 38057664

[25] Gahr, S., G. Perinetti Casoni, M. Falk-Paulsen, G. Maschkowitz, Y.T. Bryceson, and M. Voss. 2023. Viral host range factors antagonize pathogenic SAMD9 and SAMD9L variants. Exp. Cell Res. 425:113541. 10.1016/j.yexcr.2023.113541 36894052

[26] Peng, S., X. Meng, F. Zhang, P.K. Pathak, J. Chaturvedi, J. Coronado, M. Morales, Y. Mao, S.-B. Qian, J. Deng, and Y. Xiang. 2022. Structure and function of an effector domain in antiviral factors and tumor suppressors SAMD9 and SAMD9L. Proc. Natl. Acad. Sci. USA. 119:e2116550119. 10.1073/PNAS.2116550119 PMC8795524 35046037

[27] Rentzsch, P., M. Schubach, J. Shendure, and M. Kircher. 2021. CADD-Splice-improving genome-wide variant effect prediction using deep learning-derived splice scores. Genome Med. 13:31. 10.1186/S13073-021-00835-9 PMC7901104 33618777

[28] Sahoo, S.S., V.B. Pastor, C. Goodings, R.K. Voss, E.J. Kozyra, A. Szvetnik, P. Noellke, M. Dworzak, J. Starý, F. Locatelli, . 2021. Clinical evolution, genetic landscape and trajectories of clonal hematopoiesis in SAMD9/SAMD9L syndromes. Nat. Med. 27:1806–1817. 10.1038/S41591-021-01511-6 PMC9330547 34621053

[29] Jeffries, L., H. Shima, W. Ji, D. Panisello-Manterola, J. McGrath, L.M. Bird, M. Konstantino, S. Narumi, and S. Lakhani. 2018. A novel SAMD9 mutation causing MIRAGE syndrome: An expansion and review of phenotype, dysmorphology, and natural history. Am. J. Med. Genet. A. 176:415–420. 10.1002/AJMG.A.38557 29266745

[30] Dai, J., M. Mei, G. Li, L. Wang, and L. Wang. 2024. MIRAGE Syndrome Due to a de novo SAMD9 c.2944C > T (p.Arg982Cys) Variant: a Case Report and Relevant Literature Review. Clin. Lab. 70:167–170. 10.7754/CLIN.LAB.2023.230507 38213203

[31] Chin, X., A.V. Sreedharan, E.C. Tan, H. Wei, J.L. Kuan, C.W.W. Ho, J.C.M. Lam, T.W. Ting, and R.F. Vasanwala. 2021. MIRAGE syndrome caused by a de novo c.3406G>C (p. Glu1136Gln) mutation in the *SAMD9* gene presenting with neonatal adrenal insufficiency and recurrent intussusception: A case report. Front. Endocrinol. (Lausanne). 12:742495. 10.3389/FENDO.2021.742495/BIBTEX PMC8511671 34659124

[32] Jumper, J., R. Evans, A. Pritzel, T. Green, M. Figurnov, O. Ronneberger, K. Tunyasuvunakool, R. Bates, A. Žídek, A. Potapenko, . 2021. Highly accurate protein structure prediction with AlphaFold. Nature. 596:583–589. 10.1038/s41586-021-03819-2 PMC8371605 34265844

[33] Schubach, M., T. Maass, L. Nazaretyan, S. Röner, and M. Kircher. 2024. CADD v1.7: Using protein language models, regulatory CNNs and other nucleotide-level scores to improve genome-wide variant predictions. Nucleic Acids Res. 52:D1143–D1154. 10.1093/nar/gkad989 PMC10767851 38183205

[34] Ioannidis, N.M., J.H. Rothstein, V. Pejaver, S. Middha, S.K. McDonnell, S. Baheti, A. Musolf, Q. Li, E. Holzinger, D. Karyadi, . 2016. REVEL: An Ensemble method for predicting the pathogenicity of rare missense variants. Am. J. Hum. Genet. 99:877–885. 10.1016/j.ajhg.2016.08.016 PMC5065685 27666373

[35] Cheng, J., G. Novati, J. Pan, C. Bycroft, A. Žemgulyte, T. Applebaum, A. Pritzel, L.H. Wong, M. Zielinski, T. Sargeant, . 2023. Accurate proteome-wide missense variant effect prediction with AlphaMissense. Science. 2023:eadg7492. 10.1126/SCIENCE.ADG7492 37733863

[36] Fiorini, M.R., A.A. Dilliott, and S.M.K. Farhan. 2023. Evaluating the utility of REVEL and CADD for interpreting variants in amyotrophic lateral sclerosis genes. Hum. Mutat. 2023:8620557. 10.1155/2023/8620557 PMC11919158 40225153

[37] Bergquist, T., S.L. Stenton, E.A.W. Nadeau, A.B. Byrne, M.S. Greenblatt, S.M. Harrison, S.V. Tavtigian, A. O’Donnell-Luria, L.G. Biesecker, P. Radivojac, . 2025. Calibration of additional computational tools expands ClinGen recommendation options for variant classification with PP3/BP4 criteria. Genet. Med. 27:101402. 10.1016/j.gim.2025.101402 PMC12208618 40084623

[38] Rotenberg, N., C. Fortuno, M.J. Varga, A.C. Chamberlin, L. Ramadane-Morchadi, B.J. Feng, M. de la Hoya, M.E. Richardson, and A.B. Spurdle. 2025. Integration of protein stability and AlphaMissense scores improves bioinformatic impact prediction for p53 missense and in-frame amino acid deletion variants. Am. J. Hum. Genet. 112:1003–1014. 10.1016/j.ajhg.2025.01.012 PMC12120181 40233742

[39] Legrand, A., R. Demeure, A. Chantharath, C. Rey, J. Baltenneck, C.L.M. Gilchrist, J.L. Rocha, C.L. Loyer, L. Picard, A. Cimarelli, . 2025. Evolutionary characterization of antiviral SAMD9/9L across kingdoms supports ancient convergence and lineage-specific adaptations. Nat. Ecol. Evol. 9:2206–2222. 10.1038/s41559-025-02845-x PMC12680541 41219459

[40] Legrand, A., C. Dahoui, C. De La Myre Mory, K. Noy, L. Guiguettaz, M. Versapuech, C. Loyer, M. Pillon, M. Wcislo, L. Guéguen, . 2024. SAMD9L acts as an antiviral factor against HIV-1 and primate lentiviruses by restricting viral and cellular translation. PLoS Biol. 22:e3002696. 10.1371/JOURNAL.PBIO.3002696 PMC11221667 38959200

[41] Mou, Z., F. Zhang, M. Morales, B. Sahoo, X. Dai, and Y. Xiang. 2026. Structural mechanisms of SAMD9 autoinhibition and pathogenic dysregulation. Sci. Adv. 12:eaeg3967. 10.1126/sciadv.aeg3967 PMC13600222 42777028

[42] Sekiguchi, Y., S.S. Sahoo, M.L. Oldham, A. Nourse, M.W. Wlodarski, and R.C. Kalathur. 2026. Cryo-EM structures of SAMD9L reveal the arrangement and coordination of multi-domains. bioRxiv. 10.64898/2026.02.01.703102 (Preprint posted February 02, 2026).

[43] Hou, G., W. Beatty, L. Ren, Y.S. Ooi, J. Son, Y. Zhu, Q. Sheng, W. Huang, D. Li, C. Liu, . 2025. SAMD9 senses cytosolic double-stranded nucleic acids in epithelial and mesenchymal cells to induce antiviral immunity. Nat. Commun. 16:3756. 10.1038/s41467-025-59090-w PMC12015307 40263291

[44] Brnich, S.E., A.N. Abou Tayoun, F.J. Couch, G.R. Cutting, M.S. Greenblatt, C.D. Heinen, D.M. Kanavy, X. Luo, S.M. McNulty, L.M. Starita, . 2019. Recommendations for application of the functional evidence PS3/BS3 criterion using the ACMG/AMP sequence variant interpretation framework. Genome Med. 12:3. 10.1186/S13073-019-0690-2 PMC6938631 31892348

[45] Stranneheim, H., K. Lagerstedt-Robinson, M. Magnusson, M. Kvarnung, D. Nilsson, N. Lesko, M. Engvall, B.-M. Anderlid, H. Arnell, C.B. Johansson, . 2021. Integration of whole genome sequencing into a healthcare setting: High diagnostic rates across multiple clinical entities in 3219 rare disease patients. Genome Med. 13:40. 10.1186/S13073-021-00855-5 PMC7968334 33726816

[46] Sarthy, J., J. Zha, D. Babushok, A. Shenoy, J.M. Fan, G. Wertheim, A. Himebauch, A. Munchel, A. Taraseviciute, S. Yang, . 2018. Poor outcome with hematopoietic stem cell transplantation for bone marrow failure and MDS with severe MIRAGE syndrome phenotype. Blood Adv. 2:120–125. 10.1182/BLOODADVANCES.2017012682 PMC5787871 29365320

[47] Buonocore, F., P. Kühnen, J.P. Suntharalingham, I. Del Valle, M. Digweed, H. Stachelscheid, N. Khajavi, M. Didi, A.F. Brady, O. Blankenstein, . 2017. Somatic mutations and progressive monosomy modify SAMD9-related phenotypes in humans. J. Clin. Invest. 127:1700–1713. 10.1172/JCI91913 PMC5409795 28346228

[48] Pastor, V.B., S.S. Sahoo, J. Boklan, G.C. Schwabe, E. Saribeyoglu, B. Strahm, D. Lebrecht, M. Voss, Y.T. Bryceson, M. Erlacher, . 2018. Constitutional SAMD9L mutations cause familial myelodysplastic syndrome and transient monosomy 7. Haematologica. 103:427–437. 10.3324/HAEMATOL.2017.180778 PMC5830370 29217778

[49] Shima, H., M. Hayashi, T. Tachibana, M. Oshiro, N. Amano, T. Ishii, H. Haruna, M. Igarashi, M. Kon, R. Fukuzawa, . 2018. MIRAGE syndrome is a rare cause of 46,XY DSD born SGA without adrenal insufficiency. PLoS One. 13:e0206184. 10.1371/JOURNAL.PONE.0206184 PMC6221305 30403727

[50] Yoshida, M., K. Tanase-Nakao, H. Shima, R. Shirai, K. Yoshida, T. Osumi, T. Deguchi, M. Mori, Y. Arakawa, M. Takagi, . 2020. Prevalence of germline GATA2 and SAMD9/9L variants in paediatric haematological disorders with monosomy 7. Br. J. Haematol. 191:835–843. 10.1111/BJH.17006 32770553

[51] Ahmed, I.A., M.S. Farooqi, M.T. Vander Lugt, J. Boklan, M. Rose, E.D. Friehling, B. Triplett, K. Lieuw, B.D. Saldana, C.M. Smith, . 2019. Outcomes of hematopoietic cell transplantation in patients with germline SAMD9/SAMD9L mutations. Biol. Blood Marrow Transplant. 25:2186–2196. 10.1016/J.BBMT.2019.07.007 PMC7110513 31306780

[52] Nagata, Y., S. Narumi, Y. Guan, B.P. Przychodzen, C.M. Hirsch, H. Makishima, M. Aly, V. Pastor, T. Kuzmanovic, . 2018. Germline loss-of-function SAMD9 and SAMD9L alterations in adult myelodysplastic syndromes. Blood. 132:2309–2313. 10.1182/BLOOD-2017-05-787390 PMC6251008 30322869

[53] Alm, V., C. Öhberg, P. Mitev, A. Johansson, G. Ludwigs, I. Abdulkarim, A. Nolan, Y. Bryceson, R. Grankvist, B. Tesi, . 2026. Dentate nucleus hyperintensities as a clue to diagnosis in *SAMD9L-* related ataxia with gain-of-function variants. J. Neurol. Neurosurg. Psychiatry. 42951356 Published ahead of print. 10.1136/jnnp-2026-339495

