# Supplementary material for: Truncating variants reveal functional divergence of the SAMD9 and SAMD9L paralogues
Source: J Hum Immun. 2026 Nov 10;3(1):e20260095. doi: 10.70962/jhi.20260095 (PMC13750292; doi:10.70962/jhi.20260095)
Supplement: Supplementary file 1 [file jhi_20260095_sourcedatafs2.pdf]

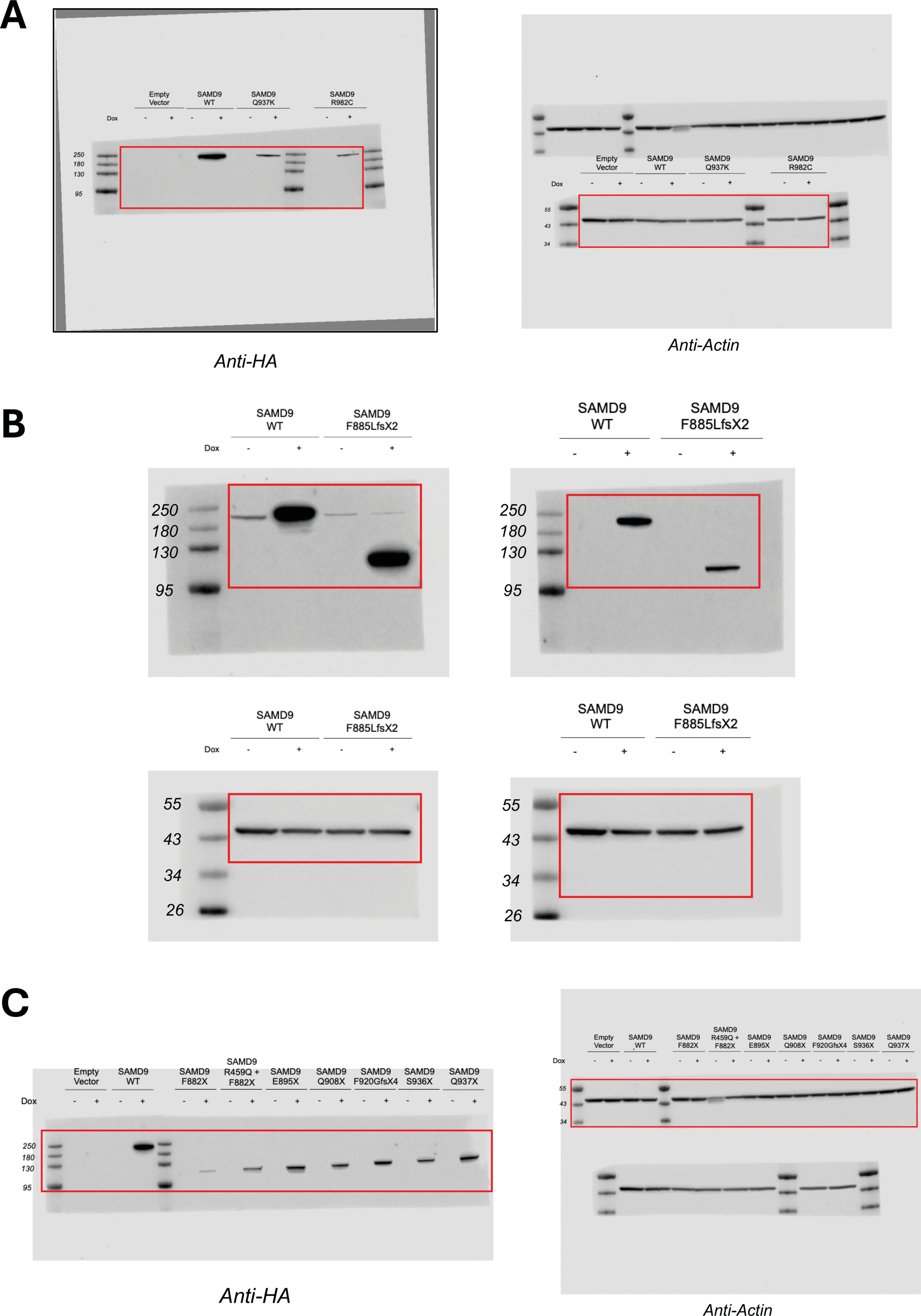

A
Empty Vector
SAMD9 WT
SAMD9 Q937K
SAMD9 R982C
Dox - + - + - + - +
250
180
130
95
Anti-HA
Empty Vector
SAMD9 WT
SAMD9 Q937K
SAMD9 R982C
Dox - + - + - + - +
55
43
34
Anti-Actin
B
SAMD9 WT
SAMD9 F885LfsX2
Dox - + - +
250
180
130
95
SAMD9 WT
SAMD9 F885LfsX2
- + - +
250
180
130
95
SAMD9 WT
SAMD9 F885LfsX2
Dox - + - +
55
43
34
26
SAMD9 WT
SAMD9 F885LfsX2
- + - +
55
43
34
26
C
Empty Vector
SAMD9 WT
SAMD9 F882X
SAMD9 R459Q + F882X
SAMD9 E895X
SAMD9 Q908X
SAMD9 F920GfsX4
SAMD9 S936X
SAMD9 Q937X
Dox - + - + - + - + - + - + - + - + - +
250
180
130
95
Anti-HA
Empty Vector
SAMD9 WT
SAMD9 F882X
SAMD9 R459Q + F882X
SAMD9 E895X
SAMD9 Q908X
SAMD9 F920GfsX4
SAMD9 S936X
SAMD9 Q937X
Dox - + - + - + - + - + - + - + - + - +
55
43
34
Anti-Actin
