# Supplementary material for: Truncating variants reveal functional divergence of the SAMD9 and SAMD9L paralogues
Source: J Hum Immun. 2026 Nov 10;3(1):e20260095. doi: 10.70962/jhi.20260095 (PMC13750292; doi:10.70962/jhi.20260095)
Supplement: Supplementary file 2 [file jhi_20260095_tables1.docx]

**Table S1. Extended clinical phenotype of patients**

| Patient | P1 | P2 | P3 | P4 | P5^†^ | P6 | P7 | P8^†^ | P9 |
| --- | --- | --- | --- | --- | --- | --- | --- | --- | --- |
| Variant Function | NoF | GoF | NoF | WT | GoF | GoF | NoF | GoF | GoF |
| SeX | Male | Female | Female | Male | Female | Female | Male | Female | Male |
| GENE  (PROTEIN VARIANT) | *SAMD9*  (p.H195R) | *SAMD9*  (p.Q937K) | *SAMD9*  (p.I1553T) | *SAMD9L*  (p.F679L) | *SAMD9L*  (p.E807K) | *SAMD9L*  (p.C883X) | *SAMD9L*  (p.W1105R) | S*AMD9L*  (p.E1176K) | SAMD9L  (p.R1281del) |
| Treatment | Antiviral treatment  (Aciclovir) | Conservative (symptomatic) | IVIGx 2 (Privigen 2 g/kg per infusion, repeated), oumedrol , prednisolon during 3 months with slow taper; anakinra (5 mg/kg/day for 2 days , discontinued due to increeased itchy rash; aspirin (discontinued) | Antiviral treatment  (Aciclovir) | Symptomatic | Antiinflammatory treatment (dexamethasone, anakinra, etoposide) followed by allogeneic HCT at 3.5 months | Symptomatic | Symptomatic |  |
| Outcome | Good | Recurrent episodes of respiratory failure and diarrhea | Complete remission; no recurrence | Sequels from encephalitis, recovered well. | Slow motor progression | Full donor chimerism. Recurrent inflammatory episodes with diarrhea and skin lesions. | Slow motor progression | Slow motor progression |  |
| Viral Infections | Chronic herpes infection | CMV viremia | SARS-CoV-2 serology positive (spike Ab >250 U/mL), PCR negative; EBV past infection; CMV negative; parvovirus negative; extended respiratory viral panel negative; fecal viral studies negative; hepatitis A and B immunity consistent with vaccination; hepatitis C negative; blood cultures negative | Varicella Encephalitis | Yes | No | No | No | No serious infections |
| Lip erosion | No | No | No | No | No | Nasal nectrotic/ inflammatory lesion | No | No |  |
| ILD | No | Yes | No | No | No | No | No | No |  |
| HSM | No | No | Splenomegaly; no hepatomegaly | No | No | No | No | No | No |
| MRI brain change | ND | Not done |  | ND | Severe cerebellar atrophy and white matter abnormalities | Extensive supratentorial with matter lesions and basal ganglia calcifications. No cerebellar atrophy. | Cerebellar atrophy | Mild cerebellar atrophy |  |
| Epiglottitis | No | No | No | No | No | No | No | No |  |
| Hemoglobin | 115-127 g/L | Low (79)* / Normal /109)*** | 121 g/L | 121-127 g/L | Reduced | Normal (181)* / Normal (132)*** | Normal | Normal | 103 |
| Platelets | 356-409 g/L | Low (191)* / Normal (389)*** | 300×10⁹/L | 217-307 g/L | Normal | Normal (178)* / Normal (268)*** | Normal | Normal | 40 |
| Neutrophils | 1.6-2.0×10⁹/L | Normal (5.0)* / Normal (8.7)*** | 8.0×10⁹/L | 2.2-2.6 ×10⁹//L | Normal | High (18.0)* / Normal (2.2)*** | Normal | Normal | 1.0 |
| Inflammation markers | Normal | Normal, but when with acute respiratory or GI symptoms – elevated ESR, CRP, sIL2R, ferritin, IgG, PLT. | CRP 44 mg/L, ESR 49 mm/h, plasma calprotectin 47 mg/L, Marked IFN-γ–driven hyperinflammation (CXCL9 44,678 pg/mL; CXCL10 >40,000 pg/mL) | Normal | Mildly elevated | Normal, but when with acute inflammatory symptoms elevated ESR, CRP, sIL2R, ferritin. | Normal | Normal |  |
| Ferritin (maximum) | ND | High (1477)** / Normal (63)*** | 9400 µg/L | 64 µg/L | Normal | High (1713)* / Normal (35)*** | ND | Normal |  |
| % B cells | 21% | Low CD19 (0.21 = 6%)* / Low (0.57 = 12%)*** |  | 16 | Normal | Low CD19 (0.03 = 1%)* / Normal (0.29 = 8%) | Normal | Normal |  |
| Bone marrow B cells | ND | 1.3% | - | ND | Hypocellularity | Not done | ND | Normal |  |
| NK cells | 0.5x109/L | Normal (0.12 = 3%)*/ Normal (0.6 = 13%)*** |  | 0.2x109/L | ND | Normal (0.27 = 7%)* / Normal (0.47 =13%)*** | ND | ND |  |
| IgG | 4.5-7.4 g/L | Normal (3.8)* / Normal (5.0)*** | 22.7 g/L | 6.9-7.3 g/L | Normal | Low (4.0)* / Normal (10.6)*** | Normal | Normal |  |
| IgA | 0.2-0.5 g/L | Normal (0.18)* / Normal (0.53)*** | 1.4 g/L | 0.7-1.0 g/L | Normal | Low (<0.08)* / Normal (1.6)*** | Normal | Normal |  |
| IgM | <0.3-1.3 g/L | High (1.8)** / Normal (1.5)*** | 1.9 g/L | 1.1 g/L | Normal | Low (<0.10)* / High (1.9)*** | Normal | Normal |  |
| T cell no. and function | Total: 4.5x109/L  CD4+: 2.9x109/L  CD8+:1.4x109/L  Good response to mitogen (PHA), Influensa, tetanus, Candida. No response to CMV, varicella and PPD. | Normal CD3 (3.08 = 89%), CD4, CD8 and proliferation* / Normal CD3 (3.59 = 74%)***, CD4, CD8 and proliferation*** |  | Total:2.7x109/L  CD4+ 1,4x109/L  CD8+ 0.9x109/L  Slightly reduced lymphocyte response to mitogen (PHA). Good response to Influenza, Tetanus Varicella.  No response to Candida, CMV and PPD. | Slightly elevated population in the bone marrow | Normal CD3 (3.35 = 91%), CD4, CD8 and proliferation* / Normal CD3 (2.71 = 77%), CD4, CD8 and proliferation*** | ND | Normal |  |
| Other | Jejunal atresia operated 2 days age. | Small for gestational age (SGA)  Possible neurodevelopmental delay.  Feeding and swallowing difficulties. |  | Recurrent pneumonia between 3-4 years old. | Mild pyramidal signs | Intrauterine growth restriction and oligo hydroamniosis.  Syndromatic features and failure to thrive. GH deficiency and elevated HbA1c.  Motor development delay and hypotonia. Possible neurodevelopmental delay and ADHD.  Feeding and swallowing difficulties. | Nystagmus | Pain and mild pyramidal signs | Bone marrow aspiration and biopsy  Smear: Good cellularity without blast increase or obvious dysplasia. Reduced myelopoiesis, absence of megakaryopoiesis  Biopsy: Generally increased fiber content, presence of hypolobulated megakaryocytes |

ILD, interstitial lung disease; HSM, hepatosplenomegaly, *) at diagnosis **) at diagnosis with ongoing infection ***) at latest follow-up.

† Additional clinical details, including the neurological phenotypes, of patients P5 and P8 are described in (53).
