# Supplementary material for: Truncating variants reveal functional divergence of the SAMD9 and SAMD9L paralogues
Source: J Hum Immun. 2026 Nov 10;3(1):e20260095. doi: 10.70962/jhi.20260095 (PMC13750292; doi:10.70962/jhi.20260095)
Supplement: Supplementary file 3 [file jhi_20260095_tables2.docx]

**Table S2. Computational pathogenicity scores and clinical annotations for *SAMD9* and *SAMD9L* variants.**

|  | **Gene** | **Variant** | **AlphaMissense Prediction** | **AlphaMissense Pathogenicity Score 0-1** | **CADD phred Score** | **REVEL Score** | **Observed Phenotype** | **Observed in patients** | **Source** |
| --- | --- | --- | --- | --- | --- | --- | --- | --- | --- |
| 1 | SAMD9 | p.Arg459Gln | likely_benign | 0.122 | 24.8 | 0.078 | GoF | Yes | (2) |
| 2 | SAMD9 | p.Asp769Asn | likely_benign | 0.119 | 18.1 | 0.257 | GoF | Yes | (2) |
| 3 | SAMD9 | p.Thr778Ile | likely_benign | 0.126 | 11.15 | 0.114 | GoF | Yes | (16) |
| 4 | SAMD9 | p.Lys821Met | likely_benign | 0.167 | 22.4 | 0.143 | GoF | Yes | (46) |
| 5 | SAMD9 | p.Arg824Gln | likely_benign | 0.086 | 15.2 | 0.035 | GoF | Yes | (29) |
| 6 | SAMD9 | p.Asn834Tyr | ambiguous | 0.383 | 24 | 0.677 | GoF | Yes | (2) |
| 7 | SAMD9 | p.Glu974Lys | likely_benign | 0.219 | 25.6 | 0.232 | GoF | Yes | (2) |
| 8 | SAMD9 | p.Arg982Cys | likely_benign | 0.238 | 15.07 | 0.152 | GoF | Yes | (47) |
| 9 | SAMD9 | p.Arg982His | likely_benign | 0.225 | 24.4 | 0.194 | GoF | Yes | (48) |
| 10 | SAMD9 | p.Ile983Ser | likely_pathogenic | 0.783 | 24.8 | 0.433 | GoF | Yes | (47) |
| 11 | SAMD9 | p.Phe1017Val | ambiguous | 0.404 | 15.9 | 0.108 | GoF | Yes | (49) |
| 12 | SAMD9 | p.Ala1195Val | likely_pathogenic | 0.597 | 24.1 | 0.251 | GoF | Yes | (2) |
| 13 | SAMD9 | p.Pro1280Leu | likely_benign | 0.319 | 10.34 | 0.118 | GoF | Yes | (2) |
| 14 | SAMD9 | p.Gln1286Lys | likely_benign | 0.111 | 14.58 | 0.013 | GoF | Yes | (2) |
| 15 | SAMD9 | p.Arg1293Trp | likely_benign | 0.166 | 23.7 | 0.123 | GoF | Yes | (16) |
| 16 | SAMD9L | p.Ser626Leu | likely_benign | 0.122 | 26.7 | 0.086 | GoF | Yes | (16) |
| 17 | SAMD9L | p.Thr665Pro | likely_benign | 0.234 | 7.294 | 0.035 | GoF | Yes | (28) |
| 18 | SAMD9L | p.Glu807Lys | likely_benign | 0.233 | 24.2 | 0.59 | GoF | Yes | (0) |
| 19 | SAMD9L | p.His880Gln | ambiguous | 0.489 | 23.7 | 0.084 | GoF | Yes | (7) |
| 20 | SAMD9L | p.Ile891Thr | likely_pathogenic | 0.720 | 26 | 0.592 | GoF | Yes | (8) |
| 21 | SAMD9L | p.Arg986Cys | likely_benign | 0.142 | 21.7 | 0.238 | GoF | Yes | (8) |
| 22 | SAMD9L | p.Arg986His | likely_benign | 0.143 | 26.5 | 0.394 | GoF | Yes | (8) |
| 23 | SAMD9L | p.Glu1176Lys | likely_benign | 0.116 | 21.3 | 0.048 | GoF | Yes | (0) |
| 24 | SAMD9L | p.Trp1180Arg | ambiguous | 0.406 | 8.031 | 0.04 | GoF | Yes | (16) |
| 25 | SAMD9L | p.Arg1281Lys | likely_benign | 0.105 | 7.86 | 0.019 | GoF | Yes | (16) |
| 26 | SAMD9L | p.Ser1509Arg | likely_benign | 0.302 | 22.8 | 0.068 | GoF | Yes | (50) |
| 27 | SAMD9L | p.Val1512Met | likely_benign | 0.251 | 25.2 | 0.098 | GoF | Yes | (48) |
| 28 | SAMD9L | p.Val1551Leu | ambiguous | 0.380 | 8.319 | 0.025 | GoF | Yes | (51) |
| 29 | SAMD9 | p.Glu184Ala | ambiguous | 0.414 | 24 | 0.235 | LoF | No | (20) |
| 30 | SAMD9 | p.Asp192Ala | likely_benign | 0.259 | 23.4 | 0.121 | LoF | No | (20) |
| 31 | SAMD9 | p.Lys198Glu | likely_pathogenic | 0.799 | 24.8 | 0.135 | LoF | No | (26) |
| 32 | SAMD9 | p.Thr205Pro | likely_benign | 0.150 | 9.408 | 0.049 | LoF | Yes | (52) |
| 33 | SAMD9 | p.Lys214Glu | likely_pathogenic | 0.602 | 24 | 0.117 | LoF | No | (26) |
| 34 | SAMD9 | p.Glu218Ala | likely_pathogenic | 0.657 | 26.2 | 0.23 | LoF | No | (20) |
| 35 | SAMD9 | p.Arg221Glu | likely_pathogenic | 0.696 | 16.54 | - | LoF | No | (26) |
| 36 | SAMD9 | p.Asp241Ala | ambiguous | 0.478 | 24.4 | 0.115 | LoF | No | (20) |
| 37 | SAMD9 | p.Ile247Thr | ambiguous | 0.523 | 23.8 | 0.212 | LoF | Yes | (52) |
| 38 | SAMD9 | p.Lys257Glu | likely_benign | 0.105 | 24.4 | 0.055 | LoF | No | (26) |
| 39 | SAMD9 | p.Leu574Pro | likely_pathogenic | 0.921 | 21.5 | 0.357 | LoF | Yes | (52) |
| 40 | SAMD9L | p.Glu186Ala | likely_pathogenic | 0.573 | 24 | 0.299 | LoF | No | (0) |
| 41 | SAMD9L | p.Glu220Gly | likely_pathogenic | 0.785 | 24.6 | 0.395 | LoF | Yes | (52) |
| 42 | SAMD9L | p.Arg223Glu | likely_pathogenic | 0.793 | 22.8 | - | LoF | No | (26) |
| 43 | SAMD9L | p.Cys228Tyr | likely_pathogenic | 0.915 | 25.8 | 0.555 | LoF | Yes | (52) |
| 44 | SAMD9L | p.Thr233Asn | likely_pathogenic | 0.570 | 25.4 | 0.255 | LoF | Yes | (8) |
| 45 | SAMD9L | p.Gly235Ser | likely_pathogenic | 0.782 | 27.6 | 0.482 | LoF | Yes | (52) |
| 46 | SAMD9L | p.Trp333Cys | likely_pathogenic | 0.877 | 20.3 | 0.191 | LoF | Yes | (52) |
| 47 | SAMD9L | p.Trp517Arg | likely_pathogenic | 0.980 | 24.6 | 0.515 | LoF | Yes | (52) |
| 48 | SAMD9L | p.Arg843Thr | likely_benign | 0.131 | 22.8 | 0.051 | LoF | Yes | (21) |
| 49 | SAMD9L | p.Lys1008Ile | likely_benign | 0.247 | 14.97 | 0.124 | LoF | Yes | (50) |
| 50 | SAMD9L | p.Arg1524His | likely_benign | 0.192 | 16.9 | 0.031 | LoF | Yes | (0) |

0 - This paper
